# Modeling gene flow distribution within conventional fields and development of a simplified sampling method to quantify adventitious GM contents in maize

**DOI:** 10.1038/srep17106

**Published:** 2015-11-24

**Authors:** Enric Melé, Anna Nadal, Joaquima Messeguer, Marina Melé-Messeguer, Montserrat Palaudelmàs, Gisela Peñas, Xavier Piferrer, Gemma Capellades, Joan Serra, Maria Pla

**Affiliations:** 1 Plant Genetics Department, Institute for Food and Agricultural Research and Technology (IRTA), 08348 Barcelona, Spain; 2 Institute of Food and Agricultural Technology (INTEA), University of Girona, 17071 Girona, Spain; 3 External consultant, InboundCycle S.L., 08039 Barcelona, Spain; 4 Estació Experimental Mas Badia, IRTA, 17134 La Tallada d’Empordà, Girona, Spain

## Abstract

Genetically modified (GM) crops have been commercially grown for two decades. GM maize is one of 3 species with the highest acreage and specific events. Many countries established a mandatory labeling of products containing GM material, with thresholds for adventitious presence, to support consumers’ freedom of choice. In consequence, coexistence systems need to be introduced to facilitate commercial culture of GM and non-GM crops in the same agricultural area. On modeling adventitious GM cross-pollination distribution within maize fields, we deduced a simple equation to estimate overall GM contents (%*GM*) of conventional fields, irrespective of its shape and size, and with no previous information on possible GM pollen donor fields. A sampling strategy was designed and experimentally validated in 19 agricultural fields. With 9 samples, %*GM* quantification requires just one analytical GM determination while identification of the pollen source needs 9 additional analyses. A decision support tool is provided.

The development and commercial use of genetically modified (GM) organisms are subject to strict legal regulations in most countries around the world. To support consumers’ informed purchasing decisions products containing GM material have to be labelled and sold as such. Tolerance thresholds for adventitious presence of GM in conventional products have been established in different countries (e.g. 0.9% in the EU^1^). Coexistence systems need to be put in place to ensure that GM and non-GM crops can be cultivated side by side without excluding any agricultural option. Several preventive measures have been proposed to limit the adventitious presence of GM material in plant products, including the use of certified seed, spatial isolation of fields, implementation of pollen barriers between fields, planning different flowering periods, etc. In the case of maize, with 55.2 million hectares (30%) GM varieties grown worldwide in 2014, pollen flow between adjacent fields is the most important potential source of adventitious GM occurrence. Implementation of ex-ante coexistence regulations needs to be accompanied with accurate GM quantification techniques and ex-post liability schemes in the event adventitious admixture of GM and non-GM products resulted in economic losses. In case a field had GM contents above the tolerance threshold due to pollen flow, it would be essential to identify the donor neighbor field(s) to clearly establish responsibilities. This can only be conclusively determined in the field before harvest.

Some attempts have been made to model maize pollen dispersal to predict the rate of GM presence in conventional fields associated with a given configuration; and to test scenarios that are likely to minimize cross-pollination risk. Several spatially explicit models have been developed to estimate the outcomes of pollen dispersal processes (e.g.^2^,^3^). Pollen concentration decreases upon increase of the distance to the emission source, as described in various dispersal functions^4^. However, prediction of pollen flow in real situations is challenging since dispersal is a stochastic phenomenon and depends on multiple environmental factors such as spatial configuration and climate^5^,^6^,^7^. Most published models focus on pollen dispersal at the field scale by applying either statistical^8^,^9^,^10^,^11^ or quasi-mechanistic^4^,^12^,^13^ approaches. Angevin and colleagues^2^ reported on the deterministic model MAPOD (Matricial Approach to Pollen Dispersal), especially designed to predict cross-pollination rates between maize fields in a spatially explicit agricultural landscape under varying cropping and climatic conditions. These models predict the number of GM grains in every non-GM maize ear as a function of a limited number of input variables. In consequence, great quantity of data and tedious simulations are required to obtain a precise description of gene flow distribution in the field. The authors developed the Global Index (GI) algorithm^14^. It estimates the contribution of every transgenic donor field to the total pollen flow in the conventional receptor field, on the basis of the distance and flowering coincidence, i.e. the parameters described to have the highest influence on cross-pollination (for a review, see^15^). Still, systematically and reliably obtaining these data is a difficult task (e.g. flowering coincidence, which has to be determined every season at a very specific time).

Experimental determination of the adventitious presence of GM material in non-GM maize fields is usually carried out by applying validated event-specific GM quantification methods, mainly real-time PCR (qPCR)^16^ to grain samples taken at different locations within the field of interest^17^,^18^. Allnutt and coworkers^19^ recommended the use of simple random sampling (at least 34 samples) for estimation of the rate of adventitious transgene presence. Considering that GM pollen flow within a given field is highly variable and specifically the known pollen flow diminution with increasing distance from the source^18^,^20^ we previously designed a more refined sampling approach (hereafter referred to as standard) that allows accurate measurement of adventitious GM contents in conventional fields^14^. It involves the analysis of at least 28 samples per field (distributed around the field perimeter, at various distances from the border up to the central zone), and thus its economic cost is largely above that of the yield of these fields, making it unviable.

MON810, expressing the *Bacillus thuringiensis* Cry1Ab toxin against corn borers, is the only GM maize transformation event cultivated in the EU. It coexists with conventional maize in agricultural fields in Catalonia, where the proportion of GM fields has continuously increased since 1998 in some zones with high corn-borer incidence. We previously thoroughly described cross-pollination of a large receptor field by a unique MON810 field in an isolated area, and deduced a simple equation describing the cross-pollination pattern that fully explained the particular case of a single donor field^21^. Here we further developed this initial equation to consider receptor fields subjected to multiple possible donor fields and experimentally demonstrated that the new model accurately explains cross-pollination distribution within the receptor field in 33 conventional fields located in regions with 70–80% GM fields, which we monitored during 6 cropping seasons^14^,^22^. Based on the model we rationally designed and validated a simplified sampling method to reliably estimate the GM contents in a field, using a very limited number of samples. It required collection of 9 samples, thorough mixture of 8 of them and only two qPCR GM determinations. In case of liability issues, individual GM contents in all 9 samples permits identifying the donor field or, alternatively, an internal cause for the adventitious GM presence in this particular field.

## Results and Discussion

### Modeling GM distribution inside receptor fields

#### Model basis: variability sources of heterogeneous GM distribution within the receptor field

Design of a reliable model requires thorough experimental-based description of adventitious GM distribution in conventional fields and identification of the main causes of cross-pollination heterogeneity. Numerous studies investigated maize pollen dispersal, both in experimental trials and real agricultural fields (for a review, see^21^,^23^,^24^,^25^,^26^). Main variability sources are associated to

##### (i) Relative geographical position of (GM) donor and (conventional) receptor fields

The mass of pollen produced in a given donor field, its intensity, diffusion and movement are crucial to explain its impact on invasion of a neighboring receptor field. Donor pollen mass is considerably larger and more dense in the immediacy of the donor field and thus its effects are stronger in the proximal than the distal borders of the receptor field (Supplementary Figure 1). In agricultural scenarios conventional fields are often surrounded by various fields, some of which sown with GM crops. This makes adventitious cross-pollination levels highly variable around the receptor field perimeter.

##### (ii) Pollen distribution within a receptor field

Adventitious pollination is a competition phenomenon that takes place between foreign pollen and that produced by the receptor field. In field sites with a unique GM pollen donor field, cross-pollination drastically decreases towards the center of the receptor field following an equilateral hyperbole pattern [1/(*d* + 1), where *d* is the distance from the field border] irrespective of overall cross-pollination percentages^21^. This suggests a common cause such as local pollen modulating pollen flow distribution. Remarkably, conventional fields subjected to multiple-source GM pollen flow showed a comparable but slightly attenuated GM diminution pattern (Supplementary Figure 2 and^14^).

##### (iii) Individual cobs

There is high cross-pollination variability between adjacent cobs subjected to the same pollen flow pressure. This was quantified in two field assays^18^,^21^,^27^ in which cross-fertilization was measured using the xenia effect, i.e. plants with yellow kernels were pollen donors whereas receptors had white kernels. Up to 904 samples were taken, each comprising 3 cobs placed at the same spot and thus subjected to the same external pollen pressure. On analysis of every cob, high SD values were observed between the 3 cobs in every group (e.g. ~0.6 for samples with cross-pollination rates about 0.9%, Supplementary Figure 3). This represents the residual variability inherent to the studied plants, and could be explained by the specific orientation, relative position between plant leaves and individual flowering dynamics of every cob.

### Model deduction and experimental validation

We modeled the cross-fertilization diminution pattern inside receptor fields. We aimed at deducing a model which did not require exhaustive data on a large number of fields every season, while being adequate to explain real agricultural situations such as putative pollen inflow from multiple donor fields. Its deduction is based on four prior assumptions:

1. Cross-pollination depends on numerous factors including the number and geographical position of donor fields, flowering coincidence, etc.^15^. Cross-pollination values around the external perimeter of the receptor field describe how external transgenic pollen inflows the conventional field to compete with self-pollen and reflect the effect of all external parameters.
2. In the event of a single external transgenic field, cross-fertilization diminution inside a receptor field follows a distribution only depending on the distance from the field border (*d*) and the cross-fertilization value at the field border (*V*_*0*_), according to the following equation: *V*_*d*_ = *V*_*0*_/(*d *+ 1), being *V*_*d*_ the cross-fertilization at a *d* distance from the field border^21^.
3. The average of the cross-fertilization values obtained at a *d* distance from the field border will follow the same diminution pattern, irrespective of possible different *V*_*0*_ values around the field perimeter. Let be the arithmetic average of values taken along the field perimeter *V*_*0,1*_, *V*_*0,2*_, *V*_*0,3*_ … *V*_*0,n*_. The corresponding values at a *d* distance will be *V*_*0,1*_/(*d* + 1), *V*_*0,2*_/(*d* + 1), *V*_*0,3*_/(*d* + 1) … *V*_*0,n*_/(*d* + 1) and the mean, *K*_*0*_/(*d* + 1).
4. Two different conventional fields with different shapes and sizes but the same area (*A*)/perimeter (*p*) ratio will have the same adventitious %*GM* in their yields when they are subjected to equivalent GM pollen flow pressure.

The model focuses on cross-fertilization means of all points equidistant from the field border: we do not consider individual spots within a field (modeling such distribution would require extensive information on the sources of the donor pollen) but concentric subareas or rings, which %*GM* means diminish at increasing *d* distances in accordance with the model’s equations.

The model is based on the concept of competition of pollen from external origin and that of the same receptor field. The total amount of external pollen is a function of the receptor field perimeter (the longest is the perimeter, the highest amount of external pollen will potentially inflow the receptor field); whereas the total amount of competing pollen produced inside a receptor field is a function of its area. A new empirical index is proposed: the field Self-Protection Index (*I*), defined as the total area and perimeter ratio (*I* = *A*/*p*). It expresses the degree of resistance to cross-fertilization by external pollen. Thus, the average %*GM* in the whole field is independent on the specific local cross-fertilization distribution, which can vary as a function of the field shape. Deduction of the model is presented in the Methods section. Crucial equations allow calculating the average %*GM* in a perimeter at a *d* distance from the field border [(9a) and (9b)], and overall %*GM* in a conventional field (12), as a function of the average value at any distance from the field border (*K*_*i*_). *K*_*i*_ summarizes the effect of all possible donor fields.

**Figure 1:**
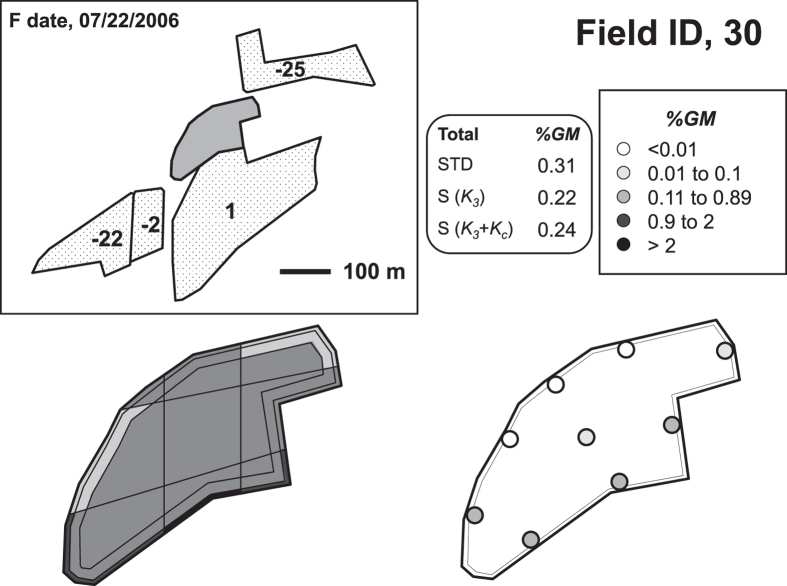
Adventitious GM contents in conventional maize agricultural field #30 in the 2006 season, taken as an example of 33 monitored fields (information on all analyzed fields is in Supplementary Figure 4 and^14^). Map showing conventional field #30, with *I* = 26.12, and the surrounding GM donor fields (up to a 120 m distance from the conventional field, represented in a dotted pattern). Central numbers correspond to the interval (in days) between male flowering of every donor field and female flowering of the receptor field (F date). Scheme below-left, adventitious GM distribution in this conventional field measured using the standard approach. Samples were taken at the intersection of the lines drawn within the field under study (i.e. at *d* = 0, 3 and 10 m around the field, and the field center) and qPCR analyzed. Colors in the scheme represent %*GM* in the different subareas (i.e. means of qPCR values at the 4 vertices of each subarea). Overall %*GM* (STD) corresponds to the weighted mean of all subareas. Scheme below-right, adventitious GM distribution in this conventional field measured using the simplified approach. The same color code is used to represent %*GM* values of *K*_*C*_ and the individual samples around the field. Overall %*GM* was calculated with *K*_*3*_ and equation (2) [S(*K*_*3*_)], and *K*_*3*_, *K*_*c*_ and equation (4) [S(*K*_*3*_ + *K*_*C*_)]. Individual analysis of the 8 periphery samples (represented in the scheme below-right) allows identifying the neighbor field causing adventitious GM occurrence (in this example, fields with flowering coincidence 1 and −2).

To test how well the model fit the experimental data adventitious %*GM* was monitored in 33 conventional field sites located in agronomic regions with 70–80% GM pressure. This ensured that most receptor fields were under the influence of multiple donor fields. Reliable %*GM* quantification was achieved by using the standard sampling strategy, which takes into account the aforementioned sources of cross-fertilization heterogeneity and is widely recognized. Results are shown in Fig. 1, Supplementary Figure 4 and^14^.

**Figure 2:**
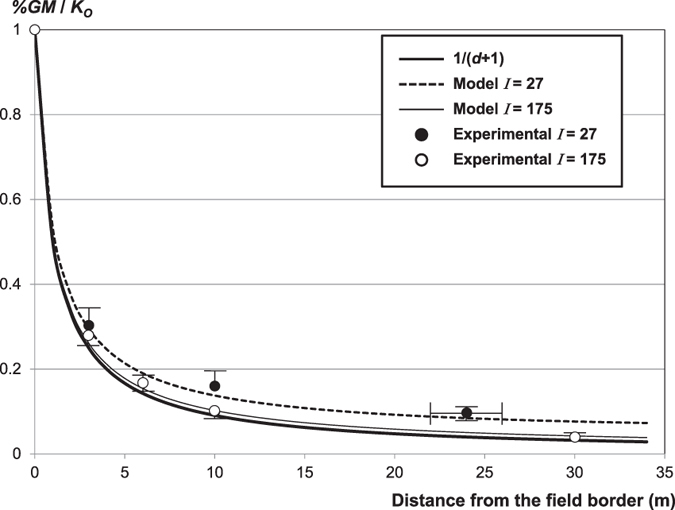
Graphical representation of 3 members of a family of curves obtained from equations (9a) and (9b) for different *I* index values; and experimental %*GM* values obtained in fields with these *I* index values. To facilitate interpretation, we used *i* = *0* in *K*_*i*_/*N*_*i*_ so that the cross-pollination value at the field border is constantly 1. Every curve corresponds to a given *I* index value (dashed: *I* = 27, thin continuous: *I* = 175); and shows mean cross-fertilization values at different distances from the border in this particular field, that is, the cross-fertilization diminution pattern. When *I* tends to ∝ the equation (9a) equals 1/(*d* + 1), here represented in a strong continuous line. Empty circles represent the experimental mean values (and standard errors) of the large field ID 1, with *I* = 175 (Supplementary Figure 4). Filled circles represent the experimental mean values (and standard errors) of six fields with *I* around 27. The distance value corresponding to the field center is represented with the standard error because it varies as a function of the field shape (and size).

Overall cross-pollination diminution patterns in these fields were distinctively extracted by global analysis of %*GM* data corresponding to samples taken at every *d* value (0, 3, 10 and around 25 m) in more than 300 available transects^14^ (transects giving 0% in all three samples were discounted because they did not add information on cross-pollination patterns). Normalization of every %*GM* data-point with the mean value in the corresponding transect gave all transects the same weight, irrespective of the actual %*GM*. There was a strong agreement between the experimental values and the theoretical curves for fields with any size and shape (*I* value), which experimentally confirms the validity of the model. As an example, Fig. 2 shows the grouped data obtained in fields with extreme *I* values: *I* = 27 ± 1 and *I* = 175.

### Design of a simplified sampling approach

Rational design of a simplified sampling strategy was carried out on the basis of the described causes for GM heterogeneous distribution in conventional fields and the model explaining the conserved pattern of GM content diminution from the edge towards the center of the receptor fields. %*GM* measurement requires the highest precision close to the legal labeling threshold, which is established 0.9% in numerous countries such as those in the EU. Fields of most sizes and shapes (*I* values from 23 to 40) with overall 0.9% GM contents have *K*_*3*_ values in the 1–0.5% range. qPCR assays for GM quantification are usually optimized and validated in this range^28^, which is covered by certified reference materials (JRC-IRMM). According to the model core (highest adventitious cross-pollination in the field border, unknown situation of possible donor fields), *K*_*3*_ should be determined from analysis of multiple samples representing the whole field perimeter. Such comprehensive sampling was unavoidable since identification of the GM pollen source (i.e. the neighboring field(s) contributing to adventitious GM presence in the field under study) was imperative for liability issues. At *d *= 3 m, samples taken close to a GM donor field have GM percentages clearly higher than those taken close to a conventional donor field (this asymmetry decreases towards the field center).

Overall %*GM* can be calculated with experimental *K*_*3*_ and *I* using formula (12 and 9b):


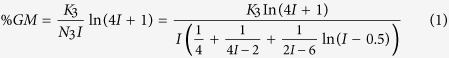


And the approximate expression [which corresponds to (1) with an error <0.02% in fields with *I* values in the 10 to 200 range, i.e. most agricultural fields]:


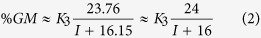


**Figure 3:**
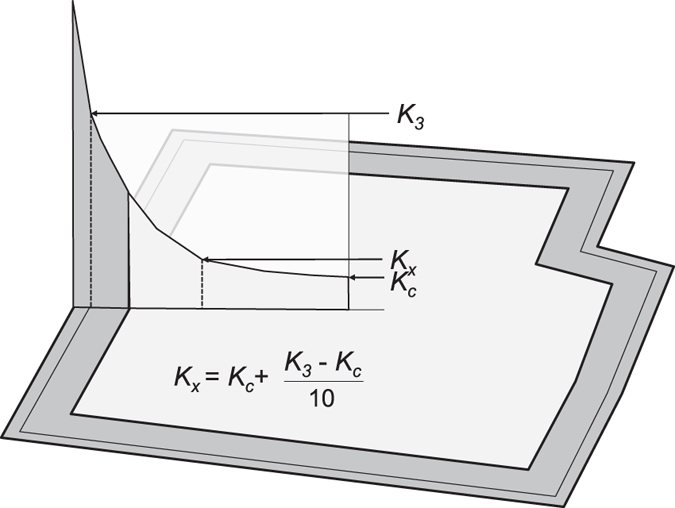
Schematic representation of cross-fertilization distribution in two concentric portions of an agronomic field: a 10 m-wide perimeter and a central portion. Estimation of the GM contents in the perimeter can be carried out on the basis of the average value 3 m inside the field (*K*_*3*_); and that in the central part on the basis of a theoretical *K*_*x*_, which depends on *K*_*3*_ and *K*_*c*_.

Cross-pollination from neighboring GM fields is not the only possible cause of adventitious GM occurrence in a conventional field. Seed admixtures or GM volunteer plants from previous cultures can entail adventitious GM presence in the yield. The sampling method should identify these situations. Analysis of an additional sample taken in the field center (*K*_*c*_) adds information on the source of adventitious %*GM* and provides increased accuracy. We propose formula (3) to calculate the overall %*GM* in a conventional field using *K*_*3*_ and *K*_*c*_ experimental values (*A*_*R*_, *A*_*C*_ and *A*_*T*_, areas of the 10 m-wide ring, the central portion and the whole field, respectively. The relative *A*_*R*_ and *A*_*C*_ areas depend on the field shape and represent its resistance to cross-fertilization by external pollen). Deduction of this equation is developed in the Methods section and Fig. 3. The approximation (4) is exact for a square field and slightly deviates as a function of the field shape:


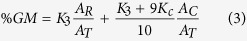



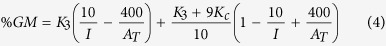


The difference between formulae (1) and (3) is illustrated with an example. Let’s imagine a rectangular field of 100 m × 150 m (*I* = 15000/500 = 30 m) subjected to a low cross-pollination pressure giving *K*_*3*_ = 0.3%. Application of the model [equation (1)] gives overall %*GM* = 0.15% [and subsequent application of equation (10) (Methods section) gives 0.06% cross-fertilization in the field center (*K*_*c*_)]. Let’s imagine this field was accidentally sown with 1% GM. Then, experimental *K*_*3*_and *K*_*c*_ values would be 1.30% and 1.06%, respectively. Using *K*_*3*_analytical value and equation (1) overall %*GM* would equal 0.65%, thus below the 0.9% threshold. In contrast, using the same *K*_*3*_plus *K*_*c*_ analytical values and equation (3) overall %*GM* would equal 1.13%, thus above the 0.9% threshold. The result of equation (3) considers the effect of both seed admixture and external pollen flow. In the absence of self-adventitious GM, equations (1, 2, 3) are highly coherent (Supplementary Figure 5).

**Figure 4:**
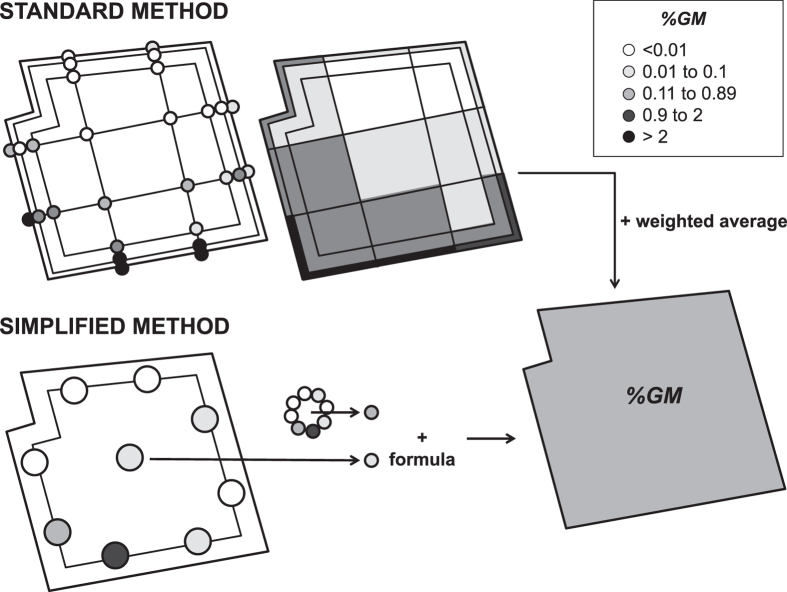
Diagram summarizing the standard sampling method (as in^14^), systematically used in numerous previous reports and in the present work; and the simplified sampling approach here proposed. The standard method consists of 28 three-cob samples taken at the intersections of 4 perpendicular transects (i.e. at *d* values around 25 m in most fields in this study); and at the intersections between these transects and three perimeters at *d* = 0, 3 and 10 m from the field edge (circles). After qPCR analysis of every sample, sub-areas are attributed a %*GM* value corresponding to the average of that of the 4 samples taken at the vertices. The field %*GM* is calculated as the weighted mean of all sub-areas. The simplified method requires taking eight 20-cob samples around the field perimeter at *d* = 3 m (it can be adapted to similar *d* values) and a central 20-cob sample. After separate grinding, an aliquot of each periphery sample are mixed to generate a single mixed sample. Only two qPCR based GM determinations are required to estimate %*GM* in the field yield using the formula (1), (3), or the approximations (2) and (4). Eight additional qPCR analyses of individual periphery samples allow identifying the source of adventitious GM. The values here depicted correspond to a real example.

A simplified sampling approach was proposed to evaluate %*GM* in a conventional field, consisting on eight samples taken equidistantly at *d *= 3 m (although it can be adapted to other distances), plus an additional sample in the field center. Due to the high cross-pollination variance in the field periphery and the expected experimental values, obtaining accurate results required large samples (e.g. 180 cobs would be needed to estimate 0.9% GM with 95% reliability, 1.95 z-statistic and ±0.1 confidence interval). We proposed 20 cobs per sample. Then, every peripheral sample is individually ground and a single mixed-sample (representing 160 cobs) is prepared with an aliquot of each: only two qPCR analyses are required to reliably assess overall %*GM* (Fig. 4). If needed, qPCR analysis of the individual periphery samples indicates the source of external GM pollen flow.

### Experimental validation of the simplified sampling method

During 5 seasons a total of 19 agricultural sites were identified in which fields sown with conventional maize were close to GM fields. Monitoring of their adventitious %*GM* using the standard and the simplified sampling methods (Table 1) showed the two approaches had a good degree of agreement (R^2^ = 0.97) (Supplementary Figure 6a). However, the slope of this regression curve was 0.82, indicating either underestimation of the %*GM* with the simplified approach, 18% overestimation with the standard method or any intermediate situation. The mathematical bases supporting the two models explain this discrepancy and show that the simplified approach fundament more accurately represents the real cross-fertilization distribution in a maize field (Supplementary Figure 6b).

As an additional control, %*GM* results obtained with the simplified method on the basis of *K*_*3*_ were compared to those using *K*_*3*_ and *K*_*c*_ (Table 1), and R^2^ was 0.97. This further confirmed the suitability of the two protocols to assess %*GM* in a field when no adventitious GM of internal origin is expected.

### Decision Support Tool

**Figure 5:**
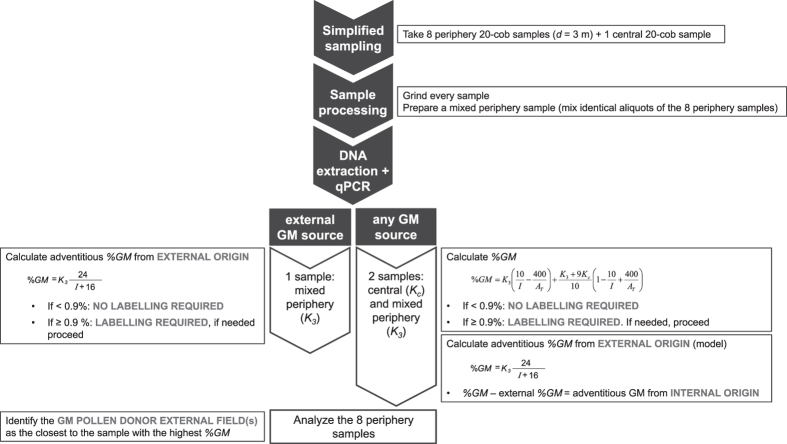
Flow chart recommended to support the analysis of adventitious GM contents in agricultural fields. Note that *I* = *A*_*T*_/*p* where *A*_*T*_ is the field area and *p* its perimeter.

In case a regulation was established that entailed coexistence control by the Competent Authorities, or farmers would choose to have an estimation of the situation of their own fields, a simple, economic and reliable method would be desirable. The simplified sampling method here designed and validated fulfills these requirements (only 1–2 analytical determinations are needed) and thus, it is a suitable option. In case of liability issues objective demonstration of the source of adventitious GM occurrence in a conventional field is relevant. Analysis of eight additional samples indicates the direction of the external pollen source. Finally, experimental assessment of %*GM* using the two simplified approaches (i.e. on the basis of *K*_*3*_; and *K*_*3*_ and *K*_*c*_) allows calculating the contribution of the field under study to overall adventitious %*GM* [by simple subtraction of %*GM* obtained with formulae (4) and (2), the latter uniquely based on GM pollen flow from external origin]. Figure 5 summarizes the proposed process in a flow chart, here adapted to the traceability and labelling EC Regulation^29^.

Additionally, the deduced equations allowed formulating several practical predictions. As an example, the maximum %*GM* (%*GM*^*max*^) in any given field can be established as a function of its *I* index using expression (12) and the maximum possible average GM cross-fertilization at *d* = 0 (*K*_*0*_^*max*^). To experimentally estimate *K*_*0*_^*max*^ a field study was set where a large donor field was immediately surrounded by a receptor field and flowering was expressly synchronized (see the scheme in^21^. As much as 140 samples, taken at *d* = 0 all around a receptor field, were analyzed using the yellow and white kernel xenia effect and the average cross-fertilization rate was 21.3%. Being MON810 maize homozygous for the transgene, the maximum possible *K*_*0*_was *K*_*0*_^*max*^ = 10.625%. Even if extremely narrow receptor fields directly encircled by synchronized donor fields could show higher *K*_*0*_values^30^ this experimental estimation can be considered as realistic in commercial fields. Similarly, a critical *I* value could be established (*I*^*thld*^) for which %*GM* from external origin is below a threshold of interest [using equation (12) with *K*_*0*_^*max*^ and the threshold %*GM*]. Fields with *I*  > 62.2 m (e.g. a square field of about 6.25 ha) will always have %*GM* levels below 0.9% in case seed admixture can be excluded; thus no specific coexistence measure is needed to commercialize their yields without the GM label in compliance with the EU legislation^1^.

Moreover, equation (11c) (Methods section) permits calculating %*GM* corresponding to the internal portion of a field. Maize harvesting usually starts by collecting the whole field periphery, and this facilitates subsequent combine maneuvers to harvest the rest of the field. Using equation (12) we deduced that, after separating the yield of a 3 m-wide periphery ring, any field with either *I* > 43 or *K*_*3*_ < (1 + *I*)/22 will have %*GM* below 0.9%. In a real example, the yield of regular-shaped field (160 × 190 m) of about 3 ha (*I* = 43), heavily affected by GM pollen flow (*K*_*0*_ = 10.625), will be below the 0.9% threshold after separate commercialization of the 3 m wide peripheral ring (labeled as GM).

## Conclusions

Here we report on a model that precisely describes the distribution of adventitious cross-pollination within maize fields, independently of the field shape, cross-pollination magnitude and geographical location of donor fields. It satisfactorily explains the %*GM* diminution pattern from the edge towards the interior of the field, both in trials designed to have a single donor field and in real coexistence conditions. We modeled mean %*GM* values of points equidistant to the field border. Thus, punctual %*GM* values cannot be estimated with the model but different mean %*GM* values can be easily integrated to estimate the overall GM content of the field (or any concentric area within).

A simplified sampling method was designed based on the model, and experimentally validated in 19 agricultural field sites during 5 seasons. If internal GM adventitious presence can be excluded (by use of certified seeds, monitoring of GM volunteer plants and control of agricultural devices and practices), analysis of one single sample allows estimation of overall %*GM* with a simple equation %*GM *≈ *K*_*3*_ (24/*I* + 16). However, up to 9 samples are taken to permit identification of the responsible GM donor field(s) and quantification of adventitious %*GM* produced inside the field.

## Methods

### Description of the studied fields

A total of 31 agricultural maize field situations were analyzed in the 2004 to 2013 seasons in the regions of Foixà, Térmens and Almacelles, Catalonia, Spain. The fields were cultivated following standard agricultural practices in the area^27^. They all consisted on a field sown with conventional maize, which was potentially under the influence of at least one field with MON810 insect-resistant GM maize. An overview of all conventional fields included in the study and additional details of every field situation and season, including GM fields surrounding every conventional field are given in Fig. 1, Supplementary Figure 4 and^14^.

### Flowering monitoring

Α set of 20 plants per field (conventional and transgenic) were tagged before flowering, and the evolution of anthesis and silking was monitored^27^. Flowering dates corresponded to the time when 50% tagged plants entered the flowering stage. Female flowering (active silks) was considered for conventional plants whereas male flowering (visible anthers shedding pollen on the tassel) was the main reference criterion for transgenic plants. Flowering synchronicity was calculated as the interval (in days) between male flowering of every donor field and female flowering of the receptor field.

### Standard sampling strategy

The standard sampling strategy^14^ (Fig. 4) consisted on 8 samples taken in the field perimeter, specifically at the intersections with 4 transects (at least 2 in perpendicular directions) crossing the field under study. In our field conditions, the distance between two of these samples was about 50–60 m. Additional transect(s) were defined in a small number of large fields. Cross-fertilization inside the receptor field was measured by additional sample points in each transect at *d* = 3 and 10 m from the perimeter; and at the crossing points between two transects (*d* ~ 25 m in these fields). Three-cob (≈1 Kg) samples were systematically collected. Note that there were 24 3-cob samples per *d* value. Experimental %*GM* values were pondered with the area they represented to calculate overall %*GM*. Note that this method copes with cross-fertilization heterogeneity by covering all field boundaries and being especially intense near the field limits.

### Determination of MON810 contents by real-time PCR (qPCR)

Sample grinding, DNA extraction and qPCR quantification of the MON810 GM event was performed as previously described^18^,^31^,^32^. For each sample, all grains were pooled and ground to a fine powder using a GRINDOMIX mill (Retsch GmbH, Haan, Germany). Maize genomic DNA was extracted and purified from 200 mg milled product using the NucleoSpin® Food kit (Macherey-Nagel GmBH, Düren, Germany) and the DNA concentration and quality was analyzed using a NanoDrop spectrophotometer (NanoDrop Technologies Inc., Wilmington DE). Specific qPCR assays targeting MON810 event^31^ and endogenous maize *adhI* gene^32^ were used. MON810 percentage was determined using relative quantification according to the standard curve approach. Standard curves for MON810 and endogenous control gene were obtained from five serial dilutions of certified reference material ERM-BF413gk 10% MON810 (Sigma-Aldrich GmBH, Munich, Germany). Results of quantification were expressed as percentages of the transgene/endogen copy ratio. Two DNA extraction and three qPCR replicates were carried out for each sample. Negative values or lack of amplification was considered for qPCR reactions with a Ct value of 50.

### Deduction of the model

Deduction of the model is based on four prior assumptions depicted in the body of the text; and the proposed Self-Protection Index (*I*).

Let’s suppose a conventional field in an area where transgenic pollen is present; and let’s suppose that *K*_*0*_ (measuring the transgenic pollen pressure around the field) is known. We will initially build a model based on *K*_*0*_ and *I*. As one of the premises is that two fields with the same *I* and *K*_*0*_ will have the same overall %*GM*, we will study into detail the simplest possible case and extrapolate the results to all other fields with the same *I*. The simplest case is a square with side *a*, in which the gene flow decreases following the 1/(*d* + 1) formula in each side.

Let’s assume that the density distribution in a square can be expressed as:


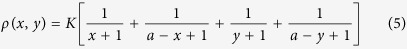


where 0 ≤ *x*, *y *≤ *a* and *K* is a proportionality constant. Note that the mean value at the border, *K*_0_, has to be determined by integrating the previous density around the border and dividing it by the perimeter. Therefore, *K* ≠ *K*_0_.

Next, we will find the average density at a given depth 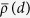. As it can be observed in Supplementary Figure 7, all points that are at a distance *d* from the field border lay in the perimeter of a concentric square with side *a-2d*. Therefore, we need to integrate the density 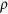 (*x*, *y*) around the perimeter of the small square, and divide the resulting value by this perimeter:


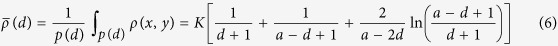


With this expression, we can find the average density at the border, 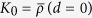, or at any other distance *i*, namely *K*_*i*_, to express the average density at a distance *i* from the border. Moreover, given that 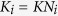, with


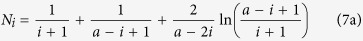


The previous equation can be rewritten in terms of *K*_*i*_ as:


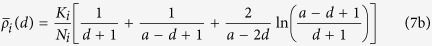


As an example, the density distribution of this field in case of a given experimental value for the density at the border *K*_*0*_, could be calculated using the previous equation with *I* = 0:


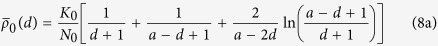


and


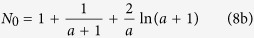


Note that at *d *= 0 we recover the value 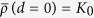.

Moreover, it’s interesting to express the previous equations as a function of *I*, which for a square field can be written as:


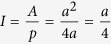


Or, what is the same, *a* = 4*I*. Therefore, the average density at a given depth, 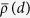, in terms of *I*, becomes:


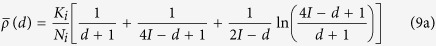



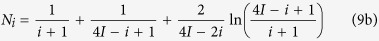


This is a crucial formula in the model because it allows predicting the average value of a perimeter at a *d* distance from the field border, and expressing it as a function of the value corresponding to the field border (using *i* = 0), or as a function of the average value at a 3 m distance from the field border (using *i* = 3).

As an example, the value at the field center (*d* = *a*/2) can be calculated on the basis of the observed value at a *d* = 3 m (this estimation will be used below):


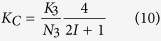


For very large fields with 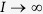 and using the *K*_*0*_ value as an example, this expression would read:


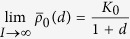


Remarkably, we recovered the initial diminution function that was the basis of the model, thus counterchecking our approach.

From the average density at a given depth 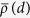, the average %*GM* density in a concentric square region 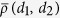 (represented in dark grey in Supplementary Figure 7) can be calculated (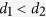):


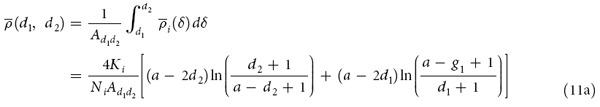


The area of the concentric region 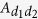 is:


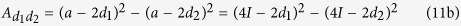


Note that equation (9a) can also be written in terms of *I*:


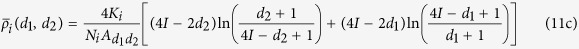


The last equation allows calculating the total average GM density in the field, %*GM*, by replacing *d*_*1*_ with 0 and *d*_*2*_ with *a*/2:


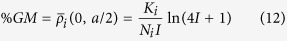


In consequence, overall GM contents in the field can be calculated using an estimation of the %*GM* in any given perimeter at a distance *i* from the field border.

### Deduction of the equations for %*GM* estimation on the basis of *K*_*3*_ and *K*_*c*_

Let’s imagine a given receptor field divided into two concentric parts: a 10 m wide perimeter and the central part (Fig. 3). Resolution of equations (9a) and (11c) with *d* = 3, *d*_*1*_ = 0 and *d*_*2*_ = 10 showed that, for fields with any *I* value, average %*GM* values 3 m inside the field approximately corresponded to the average in the 10 m wide external perimeter. This can be experimentally proved using our %*GM* dataset (see e.g. Supplementary Figure 2, where *K*_*3*_ = 0.82 and the mean value in the 10 m external perimeter equals 0.89). That is:


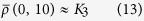


Application of equations (11c) with *d*_*1*_ = 10 and *d*_*2*_ = *a*/2 and (6) gives the following approximation for the central portion of the field:


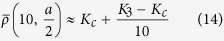


These approximations are independent on the shape and size of the receptor field. They proved to properly estimate the GM contents of the perimeter and central portion of our different shaped small and medium sized fields as compared to the standard method. For fields with *I* > 67 (i.e. a square field of about 7.3 ha) the field central portion %*GM* tends to be overestimated in about 0.0002 *I* %. This could be discounted because cross-pollination in the center of large fields is extremely rare.

In consequence, the GM contents in the 10 m perimeter can be calculated using *K*_*3*_ and those in the field center using in addition the central sample *K*_*c*_. The overall %*GM* corresponds to the weighted average of the perimeter and the central field portions. We propose formula (3) to calculate the overall %*GM* in a conventional field from the *K*_*3*_ and *K*_*c*_ experimental values, being *A*_*R*_ the area of the 10 m-wide ring, *A*_*C*_ that of the central portion and *A*_*T*_ the area of the whole field.This formula does not explicitly consider the *I* index. However, the field size and shape determine the relative *A*_*R*_ and *A*_*C*_ areas, *A*_*R*_ becoming weighty in small fields. Informatics tools are available to easily calculate these areas, e.g. those in Geographic Information Systems. As an approximation:


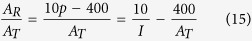


Where 10*p*-400 is *A*_*R*_. The calculation is exact for a perfectly square field, and it slightly varies as a function of the field shape. Thus, formula (3) can be expressed as formula (4).The *I* index can be calculated from the easily attainable *A*_*T*_ and perimeter.

## Additional Information

**How to cite this article**: Melé, E. *et al.* Modeling gene flow distribution within conventional fields and development of a simplified sampling method to quantify adventitious GM contents in maize. *Sci. Rep.* **5**, 17106; doi: 10.1038/srep17106 (2015).

## Supplementary Material

Supplementary Information

## Figures and Tables

**Table 1:** Compilation of the most relevant data on 19 agricultural fields sown with conventional maize, and their corresponding overall GM percentages (%*GM*) experimentally obtained using the standard and the simplified sampling methods and formula (2).

Season	Field ID	Area (m2)	*I* index (m)	*d*(m)*	*K*_d_**	*K*_C_***	%*GM*		
							SIMPLIFIED (*K*_*3*_)	SIMPLIFIED (*K*_*3*_ + K_*C*_)	STANDARD
2006	17	15,128	27.11	8	0.48	0.25	**0.51**	**0.48**	**0.64**
2006	30	14,782	26.12	8	0.24	0.05	**0.22**	**0.24**	**0.31**
2006	40	11,621	22.05	8	0.11	0.03	**0.11**	**0.12**	**0.16**
2006	105	15,870	30.94	8	0.85	0.25	**0.75**	**0.79**	**1.01**
2006	192	10,481	25.32	8	0.24	0.10	**0.25**	**0.25**	**0.41**
2007	187	22,015	35.05	6	<0.01	<0.01	**0.01**	**0.01**	**0.01**
2007	101	8,755	21.89	6	0.24	0.10	**0.22**	**0.22**	**0.17**
2007	103	7,050	12.67	6	0.11	0.05	**0.13**	**0.13**	**0.14**
2007	115	10,481	15.86	6	0.43	0.15	**0.46**	**0.47**	**0.87**
2007	192	10,473	25.3	6	1.03	0.30	**0.84**	**0.89**	**1.07**
2007	196	5,217	18.37	6	1.22	0.42	**1.16**	**1.24**	**1.44**
2007	17	15,128	27.11	6	1.94	1.31	**2.00**	**1.61**	**2.32**
2008	1	292,099	175	6	0.18	<0.01	**0.05**	**0.05**	**0.04**
2012	147	4,772	14.46	3	0.35	0.20	**0.31**	**0.29**	**0.24**
2012	149	4,829	16.15	3	0.39	0.37	**0.38**	**0.26**	**0.41**
2013	2A	2,134	6.37	3	0.01	0.01	**0.01**	**0.01**	**0.07**
2013	2B	10,766	22.57	3	0.04	<0.01	**0.02**	**0.02**	**0.01**
2013	3	16,766	26.57	3	0.06	<0.01	**0.03**	**0.03**	**0.16**
2013	4	4,232	12.02	3	0.06	0.02	**0.05**	**0.05**	**0.12**

Supplementary Figure 4 shows GM distribution within the receptor fields, as calculated in intermediate steps on application of the two methods. Note that the formula to calculate %*GM* with the simplified approach was adapted for use with the *K*_*6*_ or *K*_*8*_ (instead of *K*_*3*_) experimental data when periphery samples were taken at *d *= 8 and 6 m (2006 and 2007 seasons). The standard measure in fields with ID 187, 103 and 17 (2007) was obtained by analysis of the harvest according to the regular sampling protocols for cereals^30^. qPCR analytical values had RSD ≤ 20%. qPCR limits of detection and quantification were 0.01% and 0.3%, respectively: values between these limits must be considered approximate but were used to calculate K_*d*_ and also the %*GM* using the standard approach (which may explain some divergences in values closed to 0). Note that coexistence measures were applied in the 2013 season, which explains the low %*GM* values obtained. *sampling distance from the field border. **average GM percentage in the perimeter at the *d* distance from the field border. ***average GM percentage at the field center.
