# Supplementary material for: Modeling gene flow distribution within conventional fields and development of a simplified sampling method to quantify adventitious GM contents in maize
Source: Sci Rep. 2015 Nov 24;5:17106. doi: 10.1038/srep17106 (PMC4656998; doi:10.1038/srep17106)
Supplement: Supplementary Information [file srep17106-s1.pdf]

Supplementary Figure 1

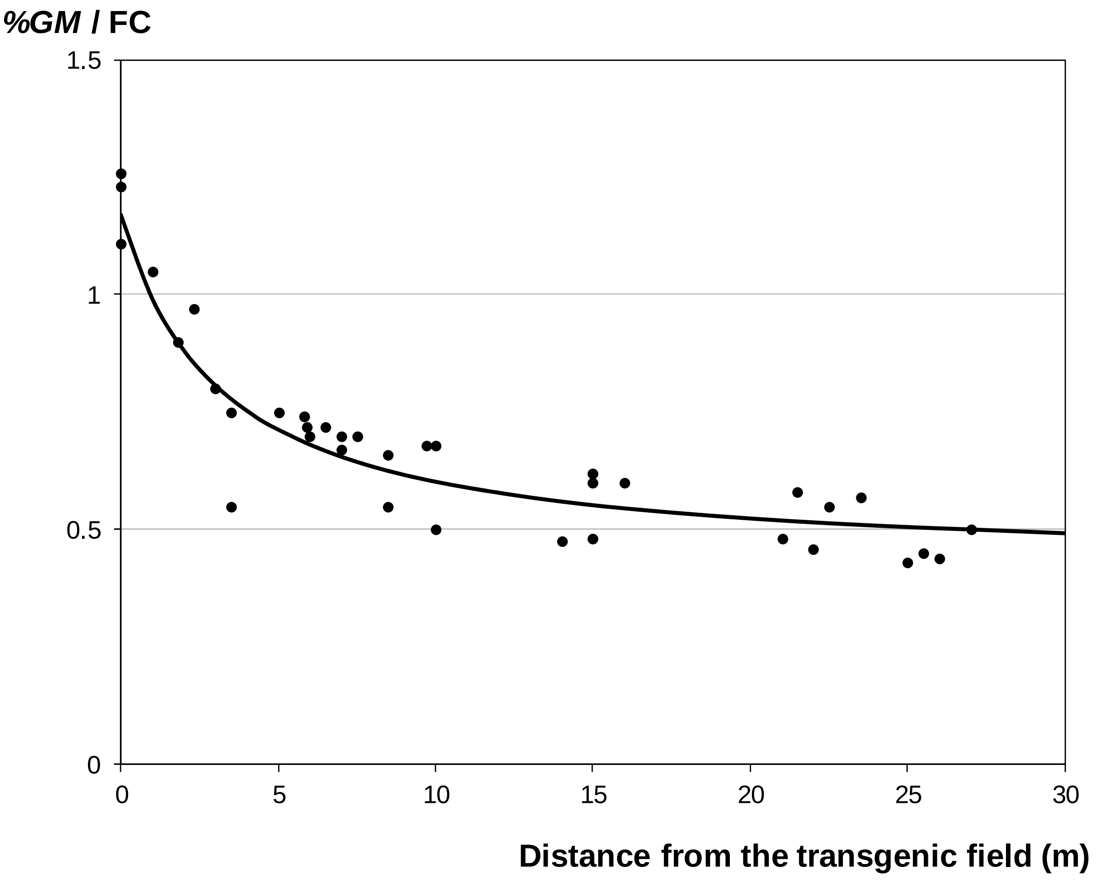

Supplementary Figure 1. GM contents at the border of conventional fields as a function of the distance to a donor GM maize field. Data on a selection of previously studied field sites[14,17], including exclusively those where conventional fields had a single GM field less than 30 apart, is shown. Every spot represents one measure in a conventional field. *%GM* (measured by means of qPCR) was normalized with flowering coincidence (days of FC) to better visualize the effect of the distance from the donor field. A tendency curve was calculated with n=36, displaying the formula 2.3/(x+3.2) + 0.42 (R^2 = 0.895). Note that the putative minor effect of GM fields more than 30 m distant from the receptor was neglected, as it was the predominant wind. Nevertheless, the experimental values obtained in a large field with one single GM pollen source[17] are in agreement with the curve.

Supplementary Figure 2

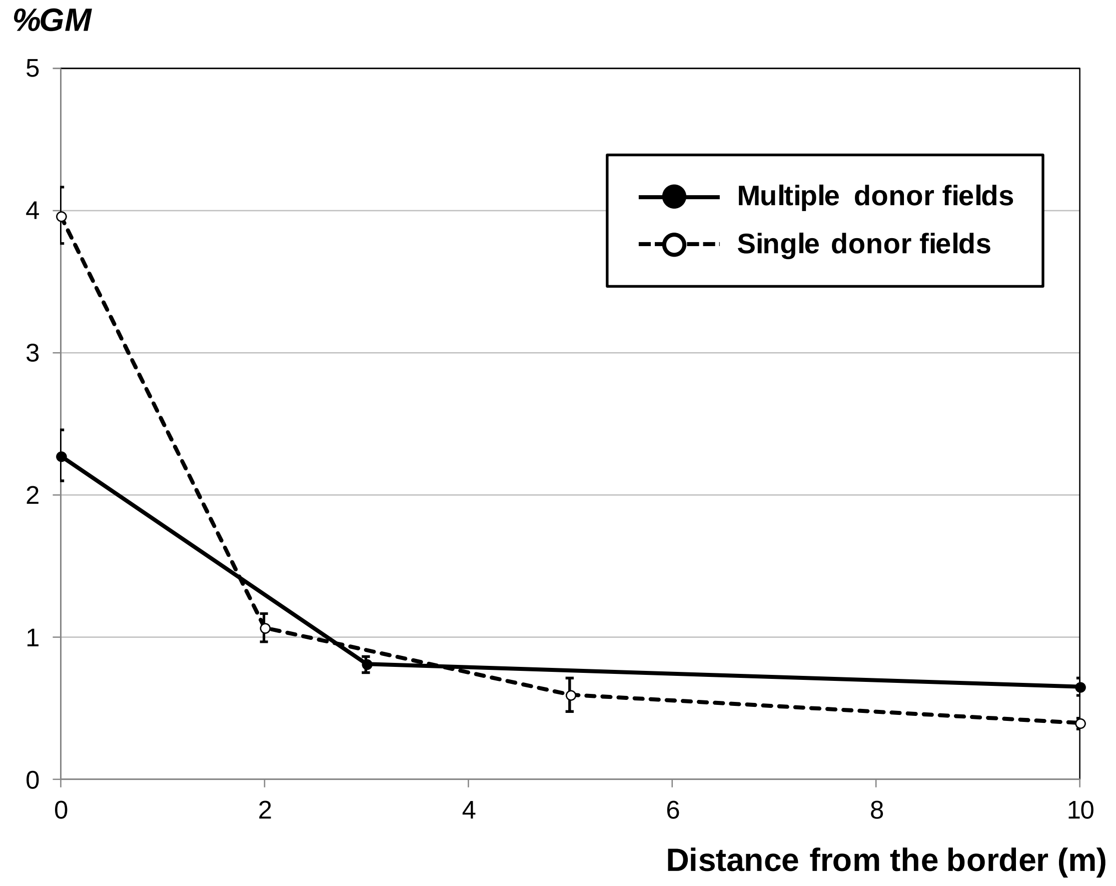

Supplementary Figure 2. Diminution of adventitious cross-pollination from the border (*d*=0) towards the center of a conventional field either subjected to multiple or a single GM pollen donor field. Continuous line: mean *%GM* (and SD) values at 0, 3 and 10 m from the field border in a total of 124 transects analyzed in conventional agricultural fields subjected to multiple GM donor fields ([14]; this work). Dotted line: mean *%GM* (and SD) values at 0, 2, 5 and 10 m from the field border in a total of 76 transects analyzed in a large field in the same area, in which there was a single GM donor field (taken from[20]). To give all transects the same relative weight, all values were normalized so that the area below the curve equals 10 (i.e. corresponding to 1% GM in the 10 m-wide external portion of every field). Fields subjected to multiple-source GM pollen flow had a slightly attenuated *%GM* diminution pattern as compared to that in fields with a single GM pollen donor. This highly relevant observation can be explained by simultaneous pollen flow from different sides of the receptor field: as we consider longer distances from one given donor field (and thus, lower cross-pollination values), they will become shorter from another donor field (and thus, higher cross-pollination values) and the real cross-pollination in this point will be dependent on the two values.

Supplementary Figure 3

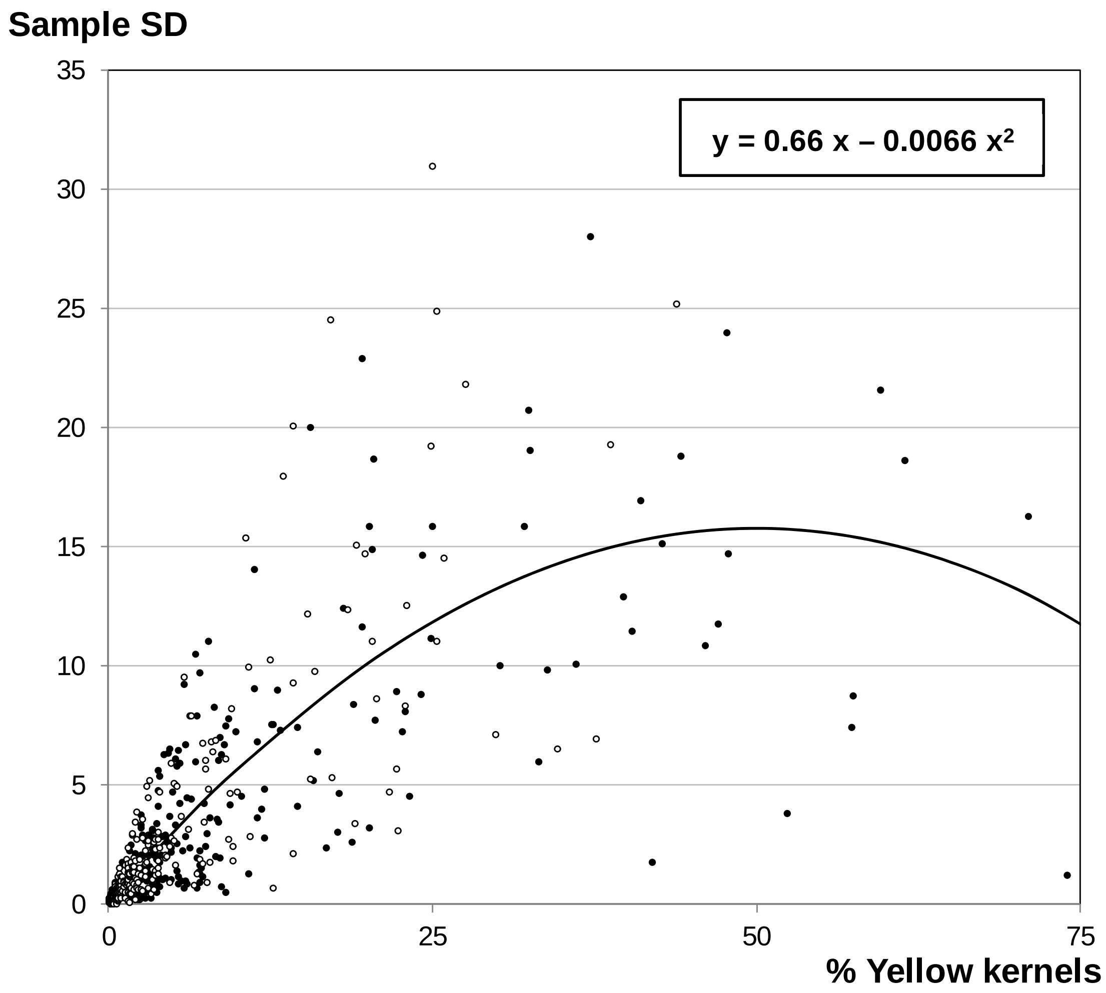

Supplementary Figure 3. Graphical representation of the SD values obtained in measuring cross-pollination in a total of 904 three-cob samples, as a function of sample mean values. The 3 cobs in every sample were taken at the same spot and were subjected to the same external pollen pressure (according to the main studied factors such as distance and flowering coincidence between donor and receptor fields, distance from the field border, etc.). Thus, the variability between these 3 cobs represents the residual variability inherent to the studied plants. The xenia effect was used to measure cross-pollination, i.e. donor and receptor plants were homozygous for the yellow (*Y*) and white (*y*) grain character, respectively. The percentages of yellow grains in receptor white cobs were obtained in two field assays: [29](filled circles) and [20](empty circles). Inset, the better fitting tendency curve is represented, which is symmetrical and crosses the x axis where y=0 and y=100, as it should be expected for a measure of percentages. The curve was calculated with the SD squares (σ), i.e. the unbiased estimator of the population variance.

Supplementary Figure 4

Field ID, 17

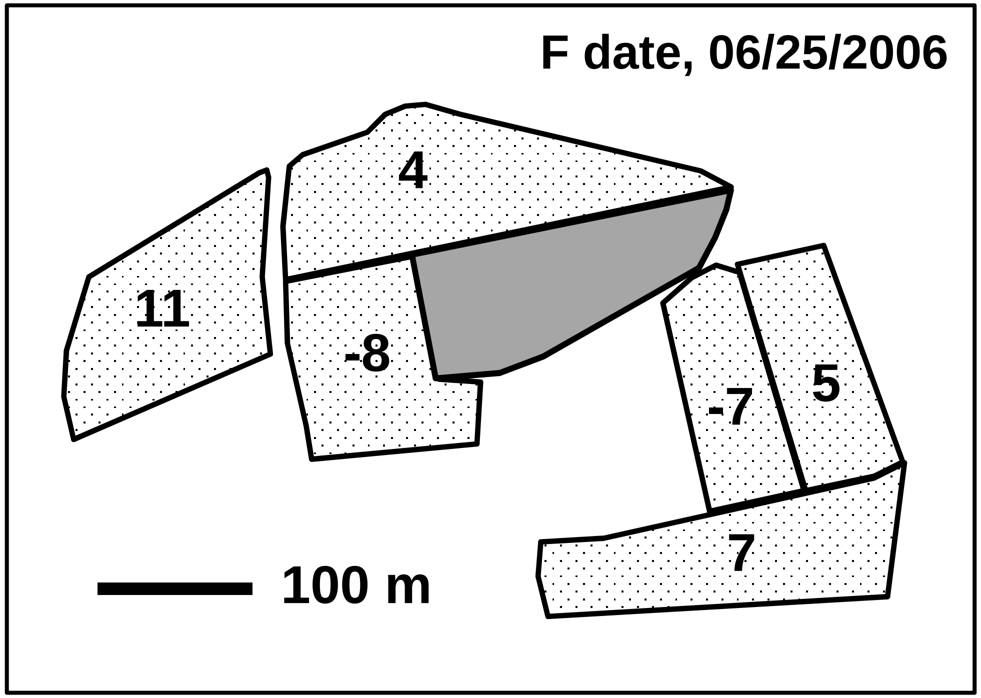

Total	*%GM*
STD	0.64
S (K_3)	0.48
S (K_3+K_c)	0.51

%GM

- <0.01
- 0.01 to 0.1
- 0.11 to 0.89
- 0.9 to 2
- > 2

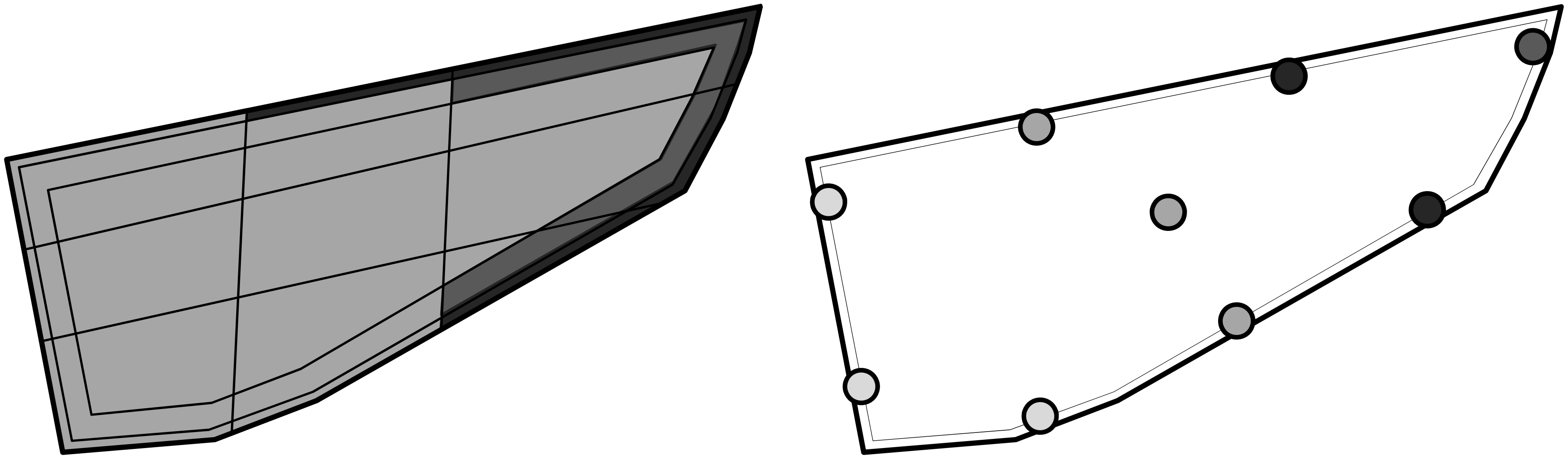

Field ID, 40

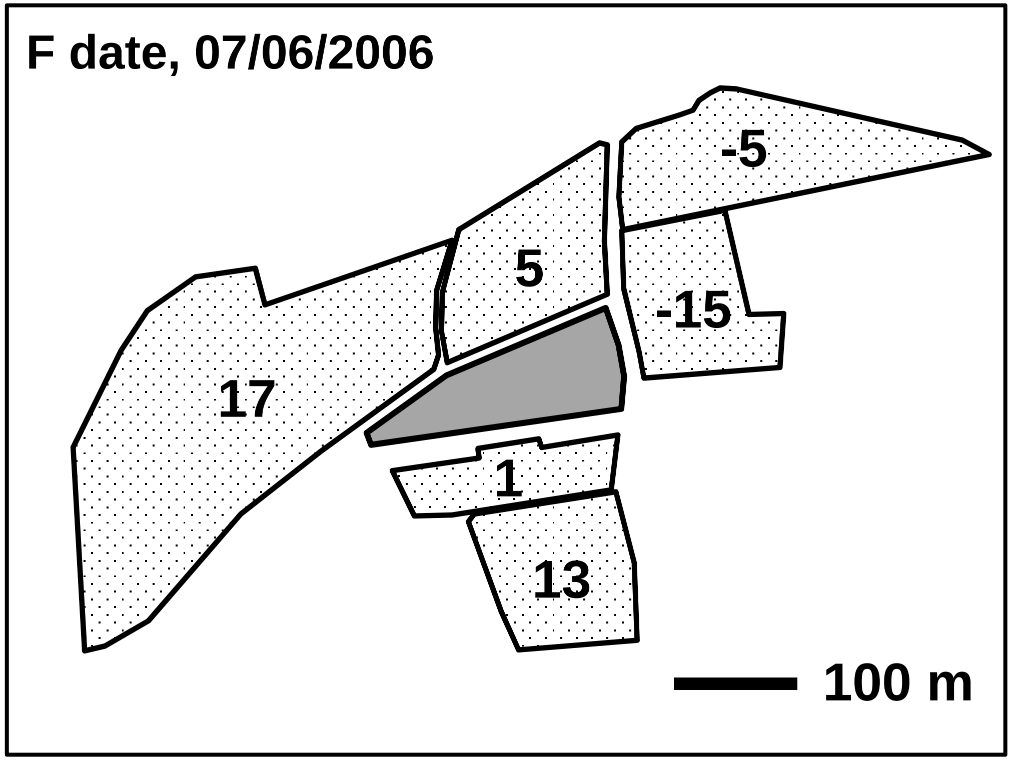

Total	%GM
STD	0.16
S (K_3)	0.11
S (K_3+K_c)	0.12

%GM

- <0.01
- 0.01 to 0.1
- 0.11 to 0.89
- 0.9 to 2
- > 2

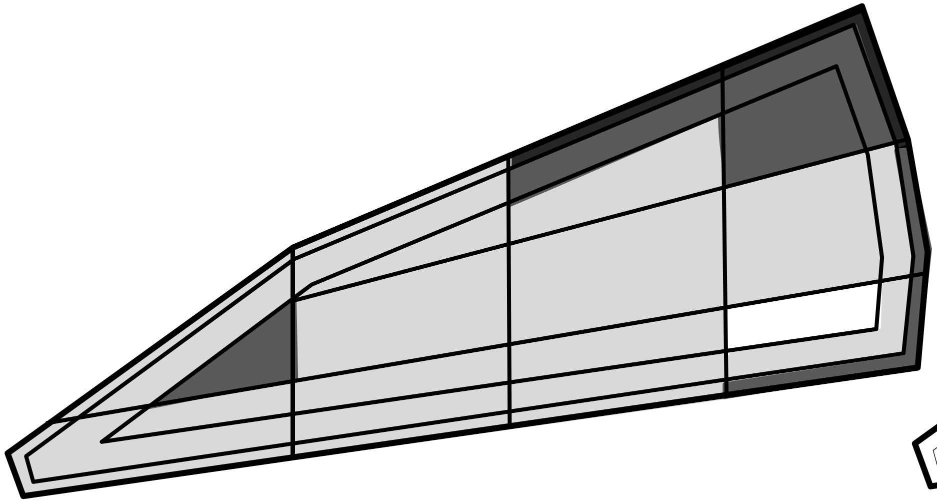

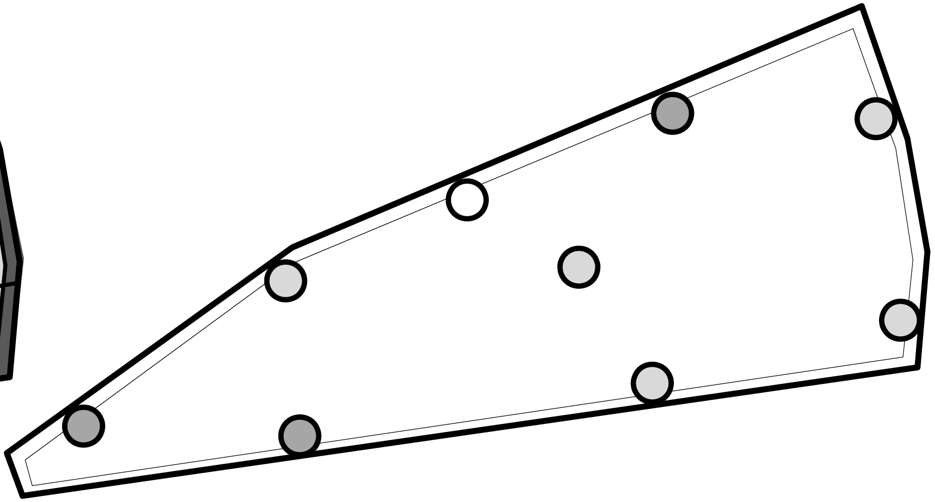

Field ID, 105

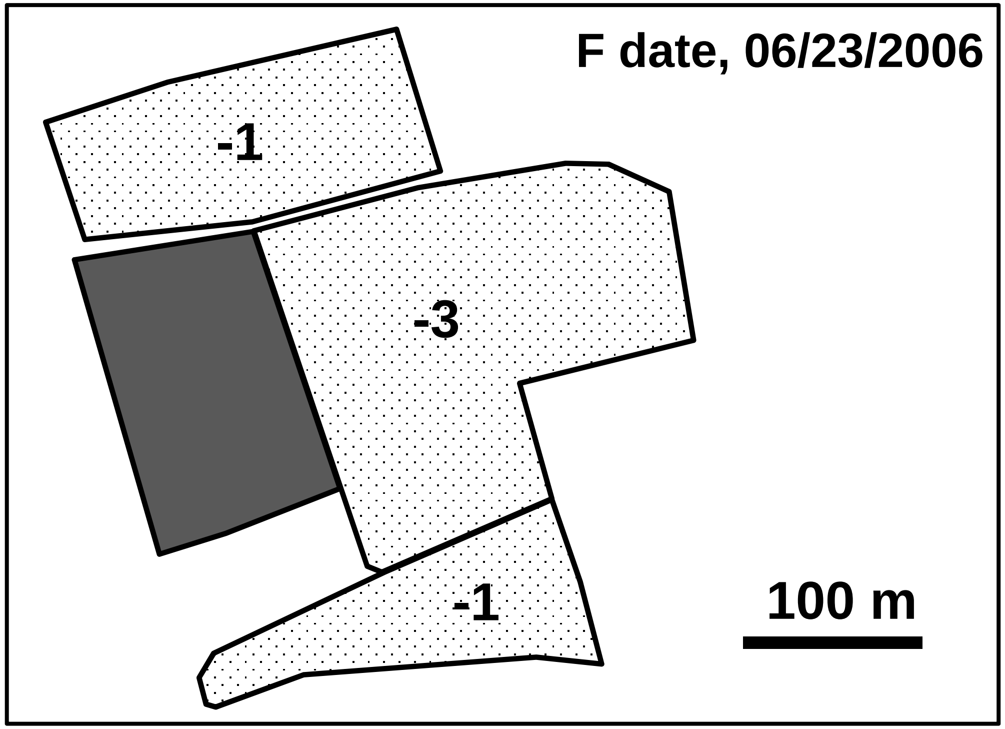

Total	%GM
STD	1.01
S (K_3)	0.75
S (K_3+K_c)	0.79

%GM

- <0.01
- 0.01 to 0.1
- 0.11 to 0.89
- 0.9 to 2
- > 2

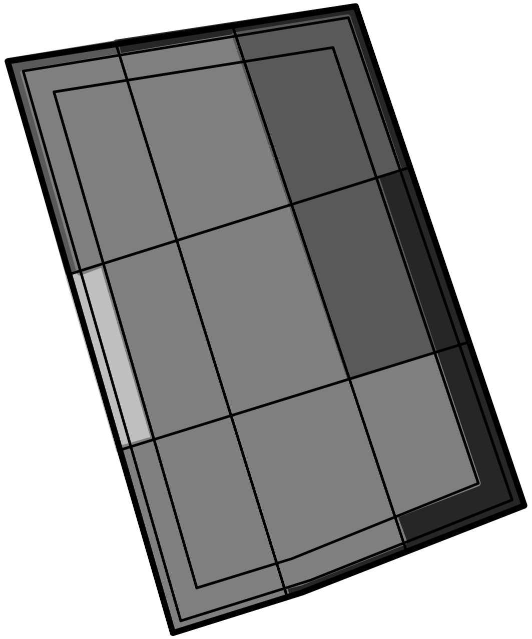

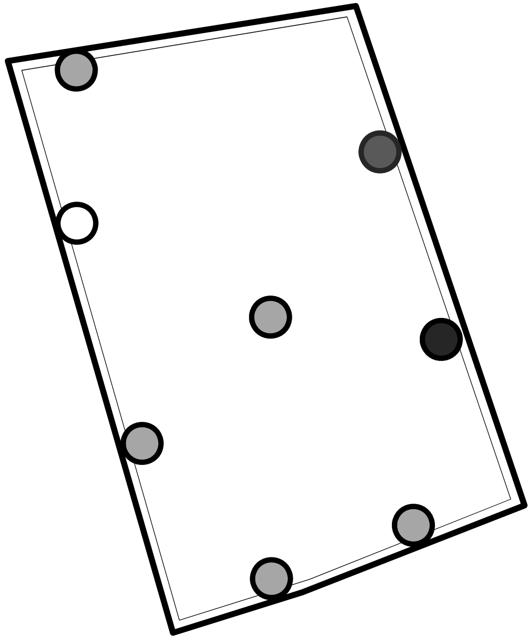

Field ID, 192

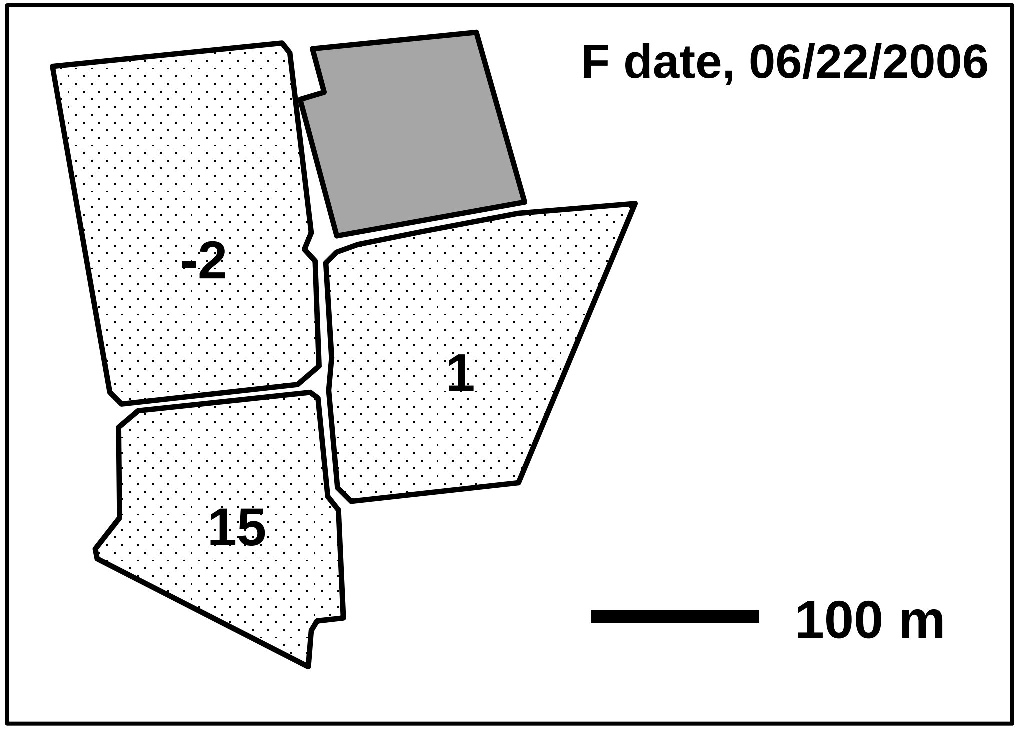

Total	%GM
STD	0.41
S (K_3)	0.25
S (K_3+K_c)	0.25

%GM

- <0.01
- 0.01 to 0.1
- 0.11 to 0.89
- 0.9 to 2
- > 2

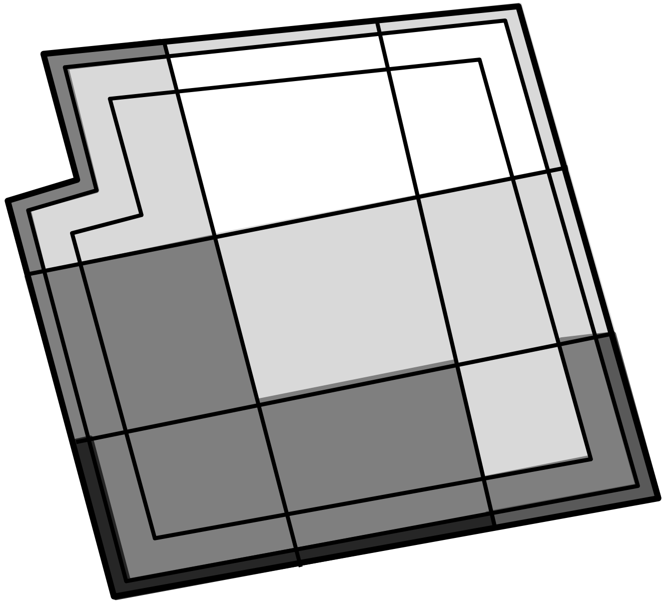

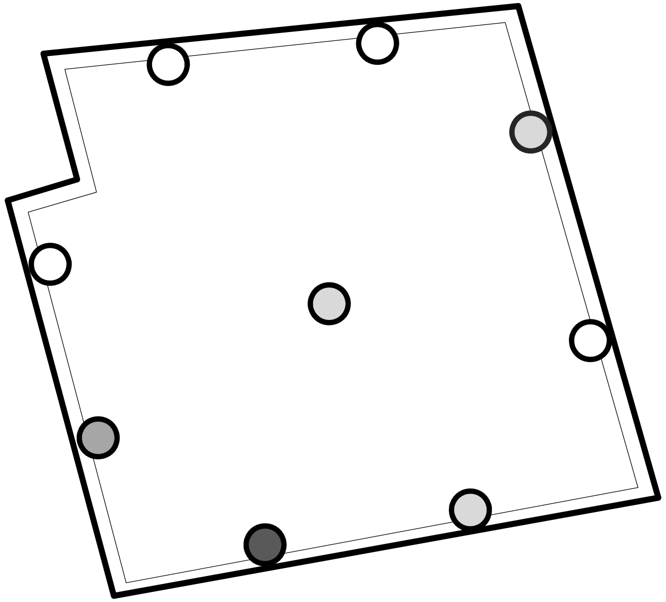

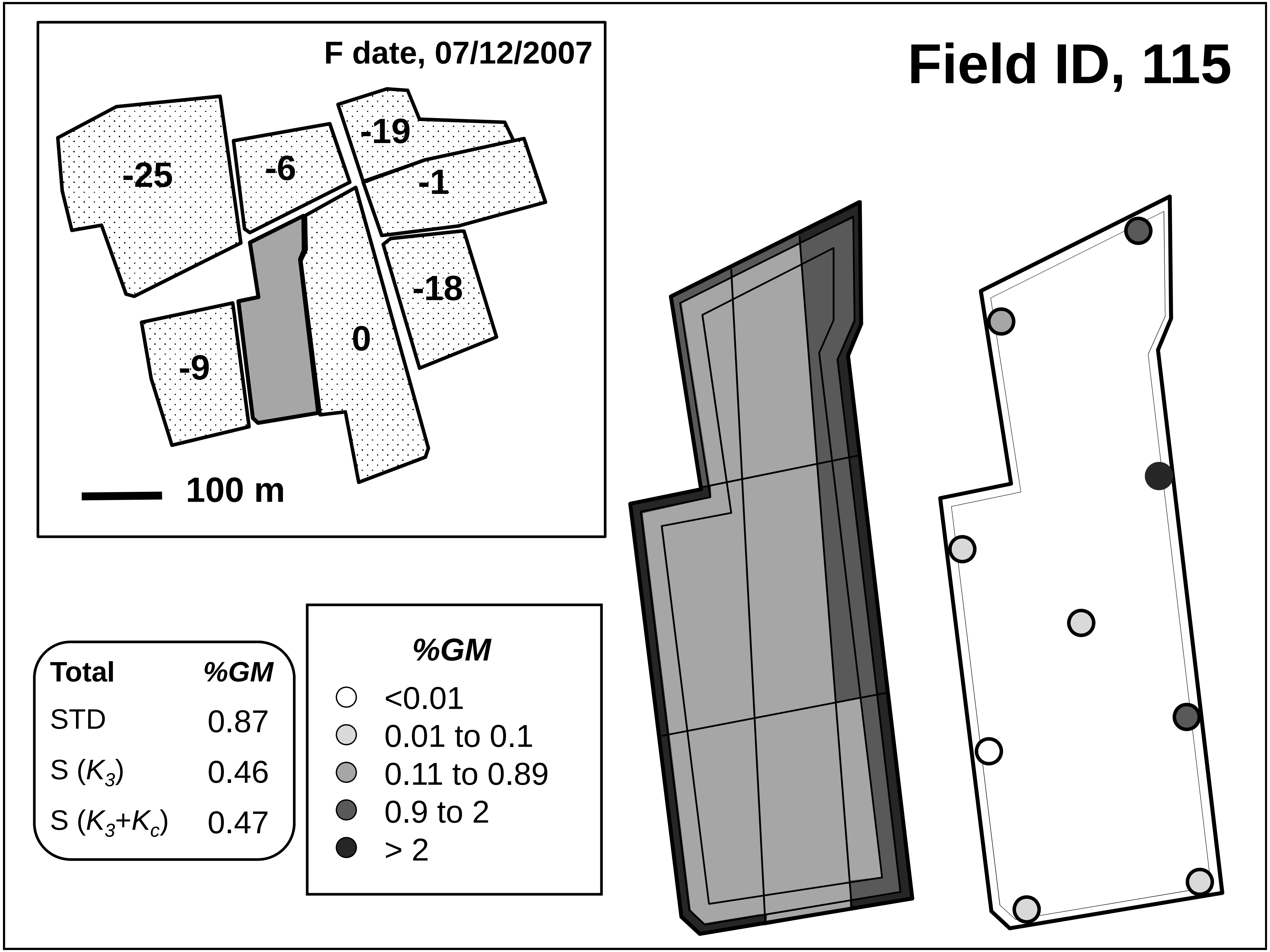
Field ID, 115
F date, 07/12/2007
-25
-6
-19
-1
-18
0
-9
100 m
Total
%GM
STD
0.87
S (K_3)
0.46
S (K_3+K_c)
0.47
%GM
<0.01
0.01 to 0.1
0.11 to 0.89
0.9 to 2
> 2

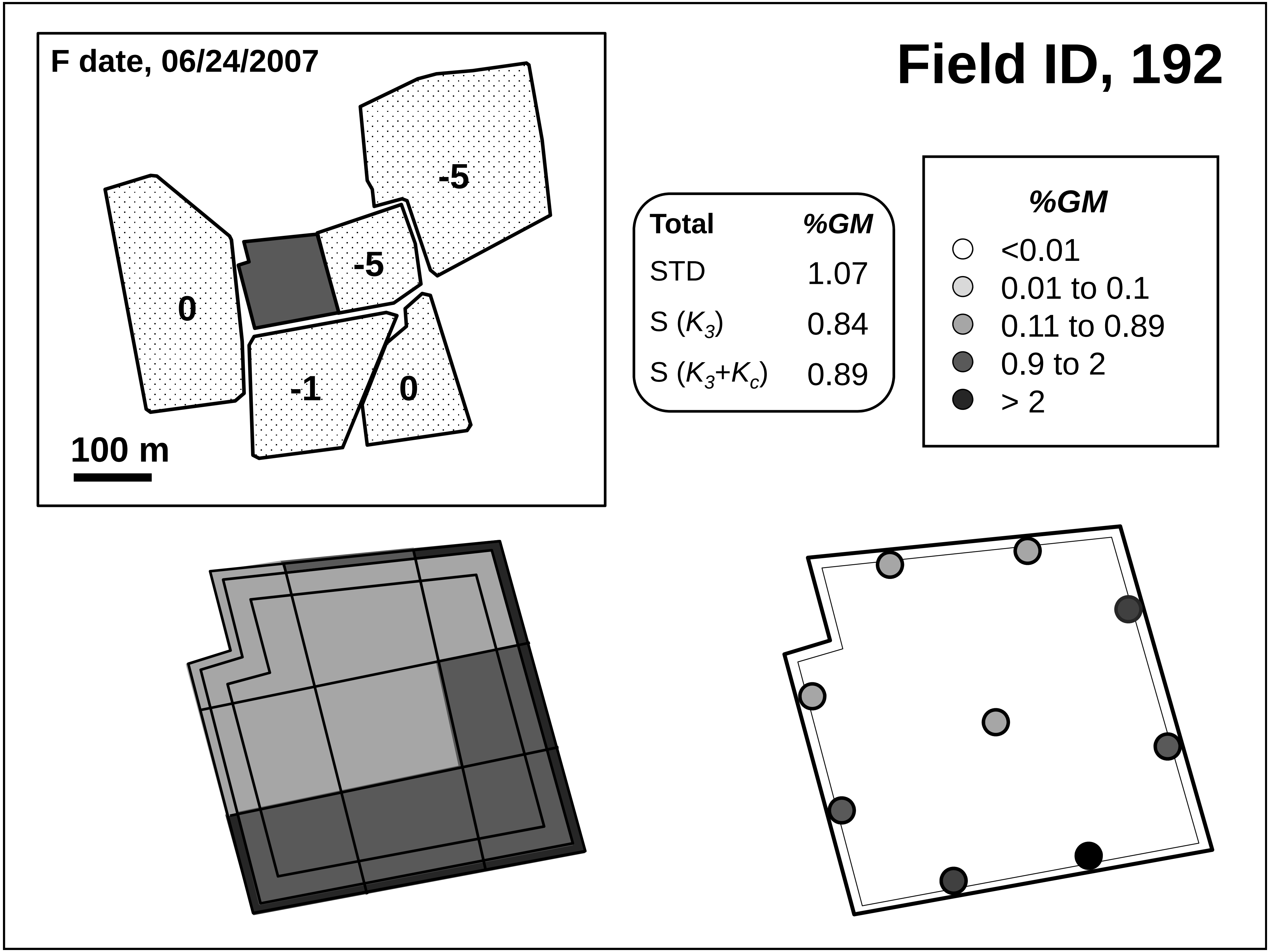
F date, 06/24/2007
-5
-5
0
-1
0
100 m
Field ID, 192
Total
%GM
STD
1.07
S (K_3)
0.84
S (K_3+K_c)
0.89
%GM
<0.01
0.01 to 0.1
0.11 to 0.89
0.9 to 2
> 2

Field ID, 187

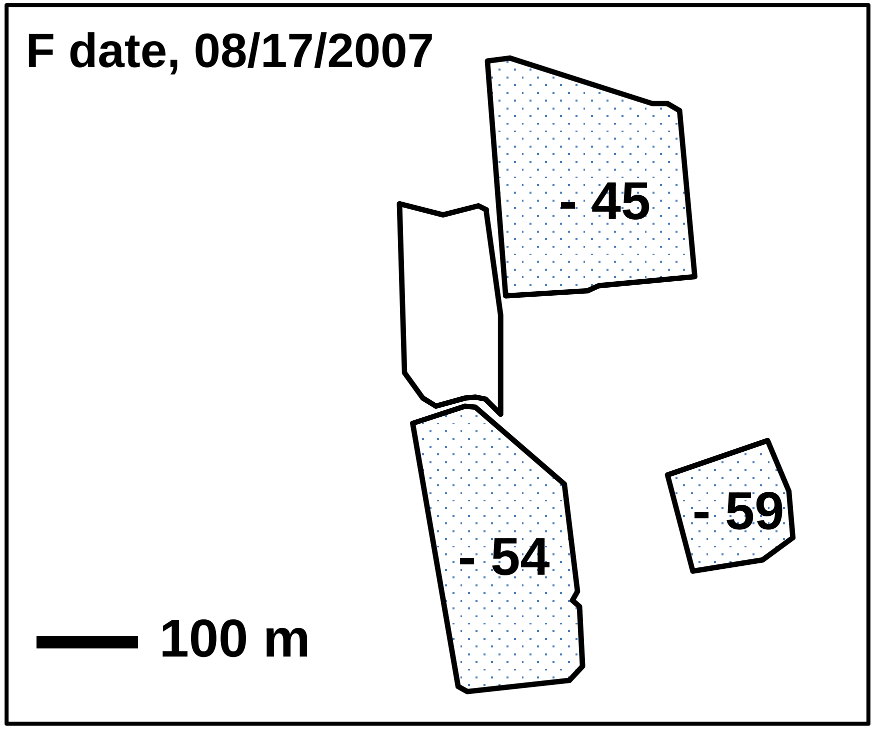

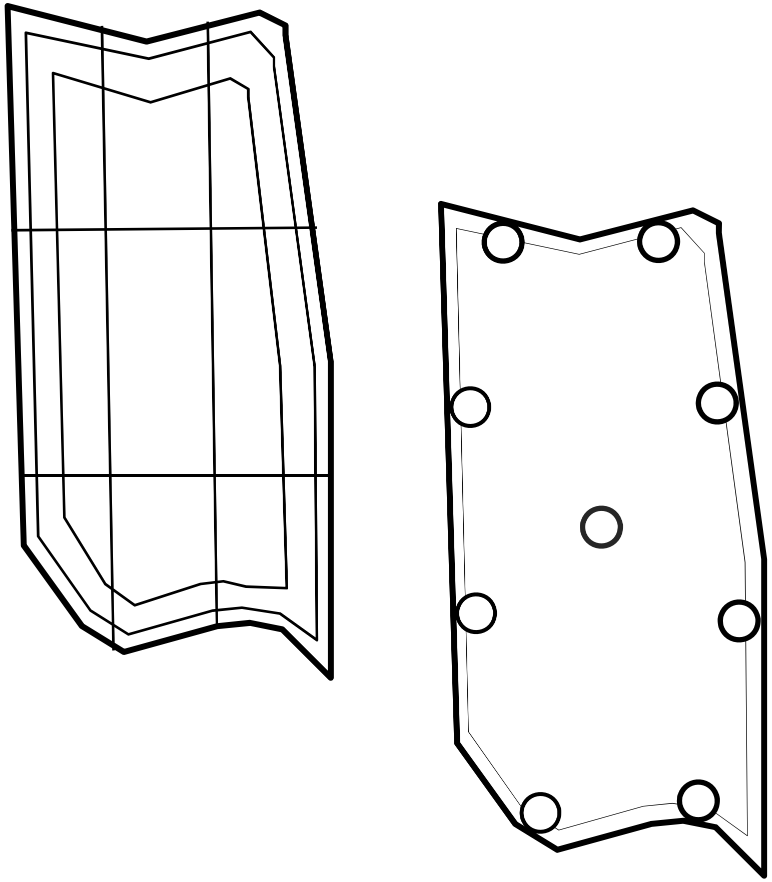

Total	%GM
STD	<0.01
S (K_3)	<0.01
S (K_3+K_c)	<0.01

%GM

- <0.01
- 0.01 to 0.1
- 0.11 to 0.89
- 0.9 to 2
- > 2

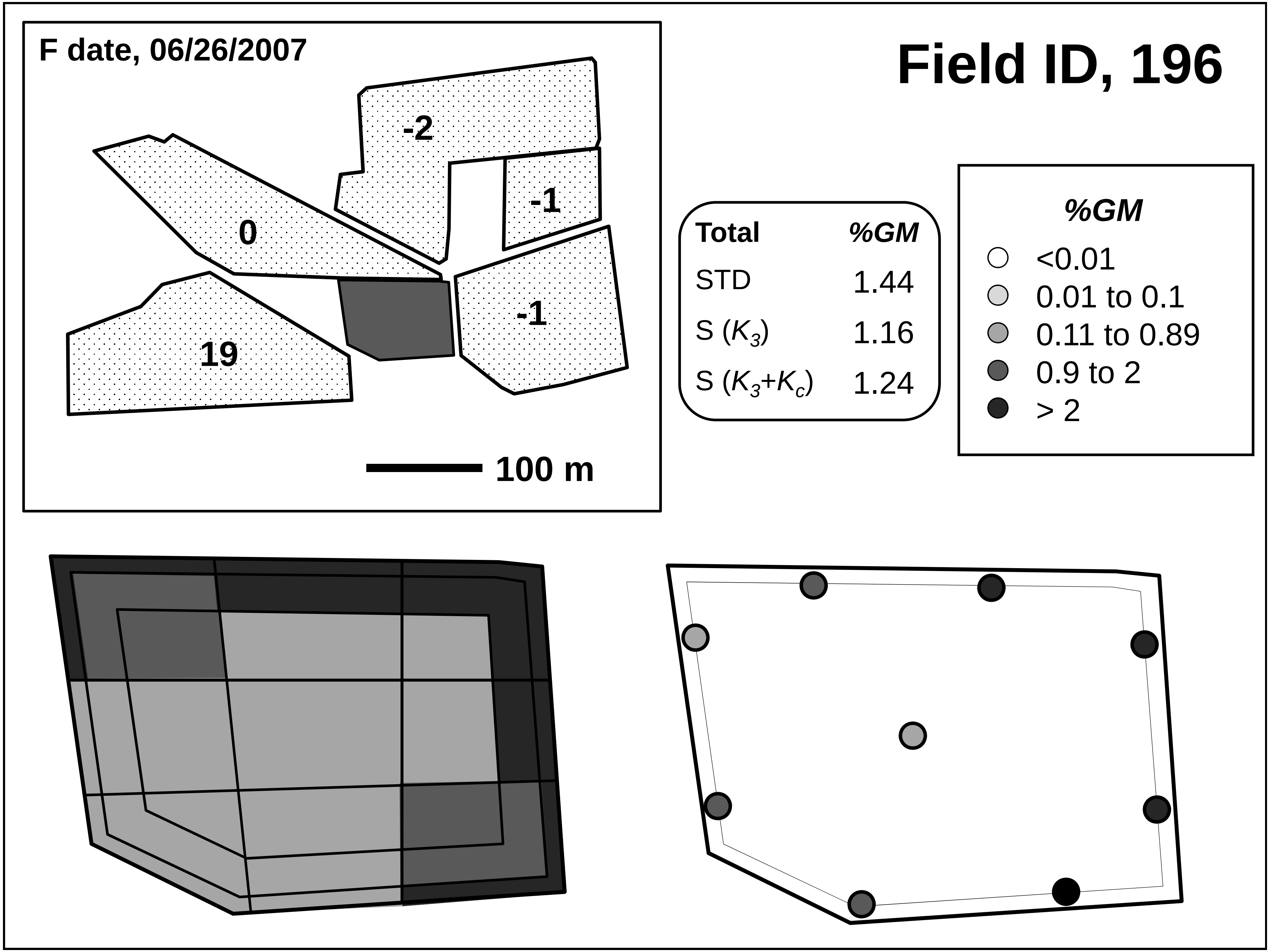

F date, 06/26/2007
Field ID, 196
-2
-1
0
-1
19
100 m
Total %GM
STD 1.44
S (K3) 1.16
S (K3+Kc) 1.24
%GM
<0.01
0.01 to 0.1
0.11 to 0.89
0.9 to 2
> 2

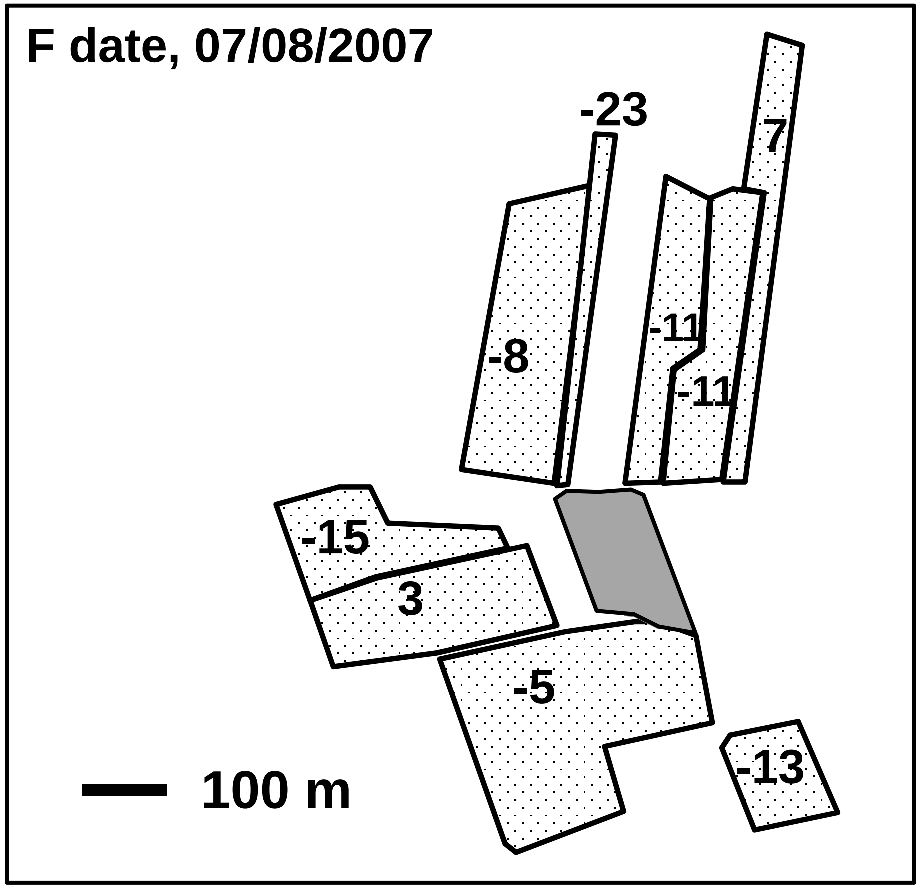

Field ID, 101

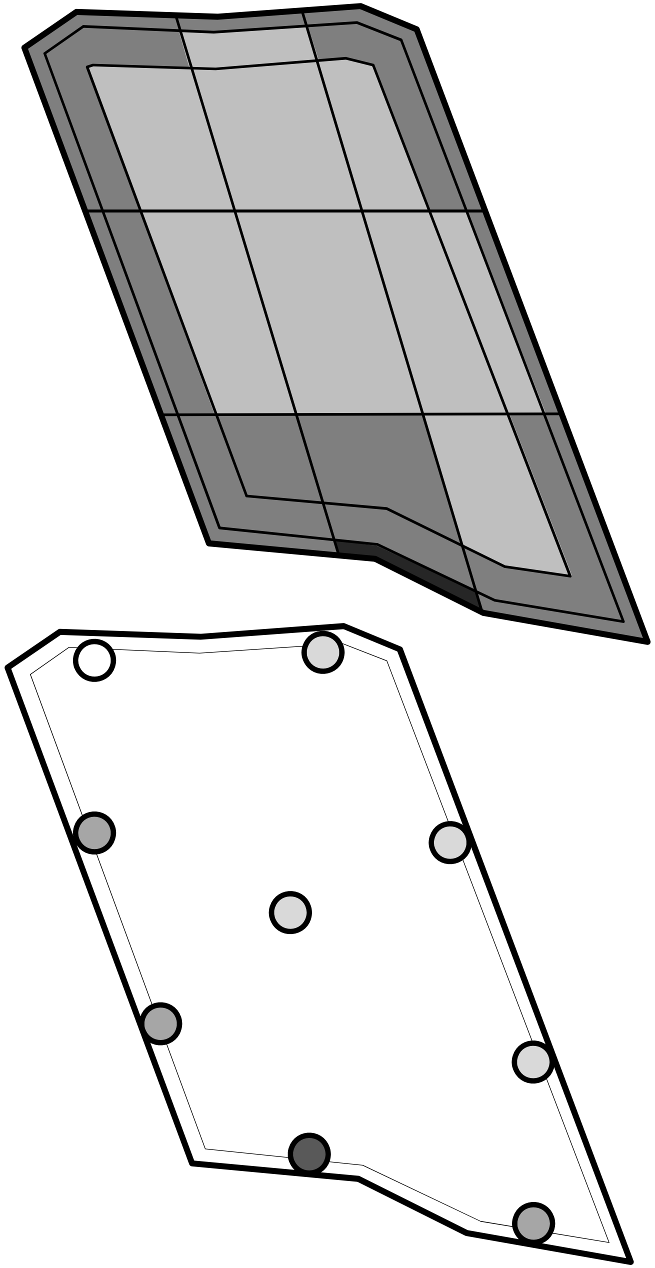

Total	%GM
STD	0.17
S (K_3)	0.22
S (K_3+K_c)	0.22

%GM

- <0.01
- 0.01 to 0.1
- 0.11 to 0.89
- 0.9 to 2
- > 2

Field ID, 17

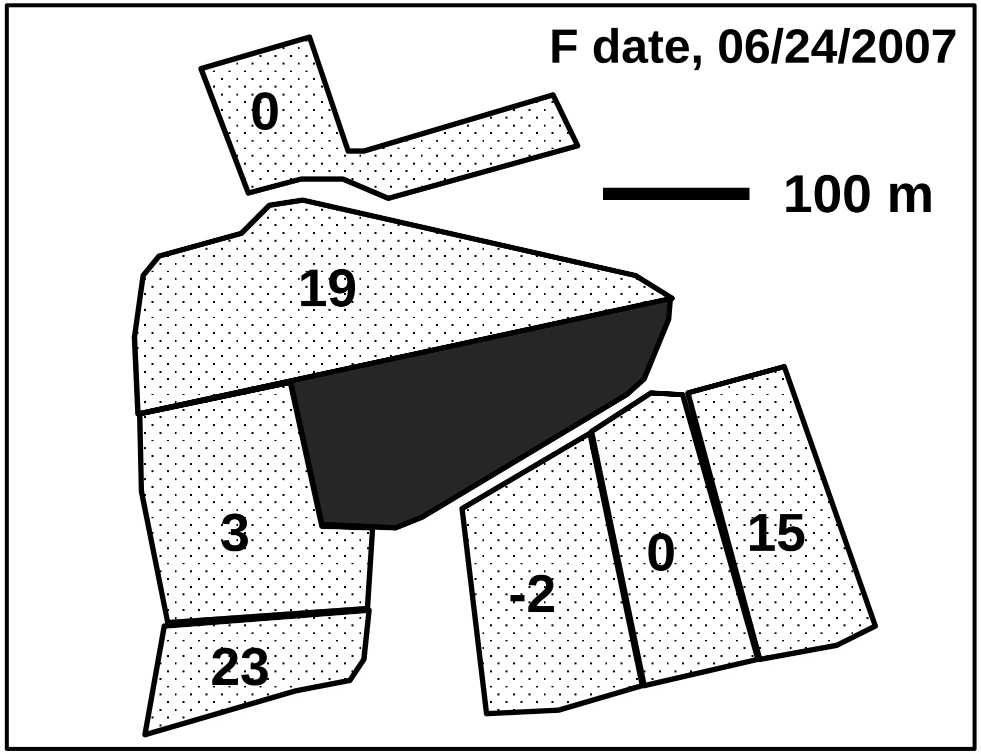

Total	%GM
STD	2.32
S (K_3)	2.00
S (K_3+K_c)	1.61

%GM

- <0.01
- 0.01 to 0.1
- 0.11 to 0.89
- 0.9 to 2
- > 2

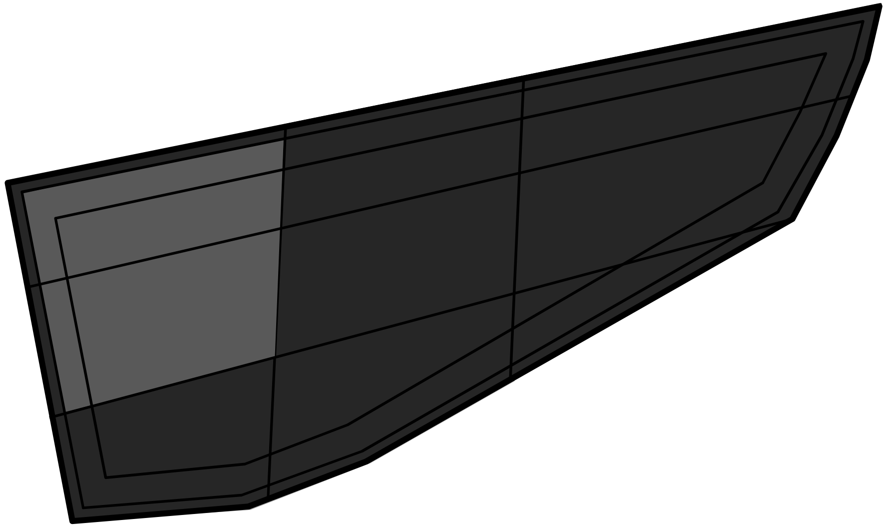

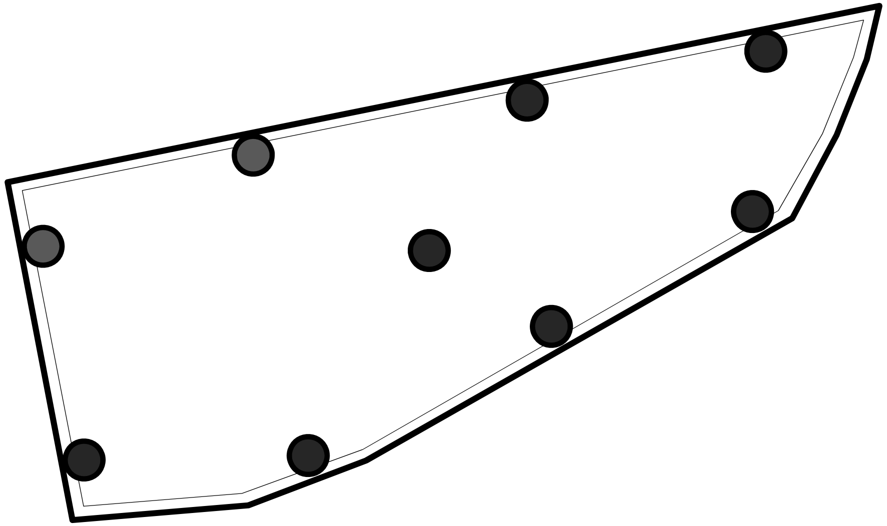

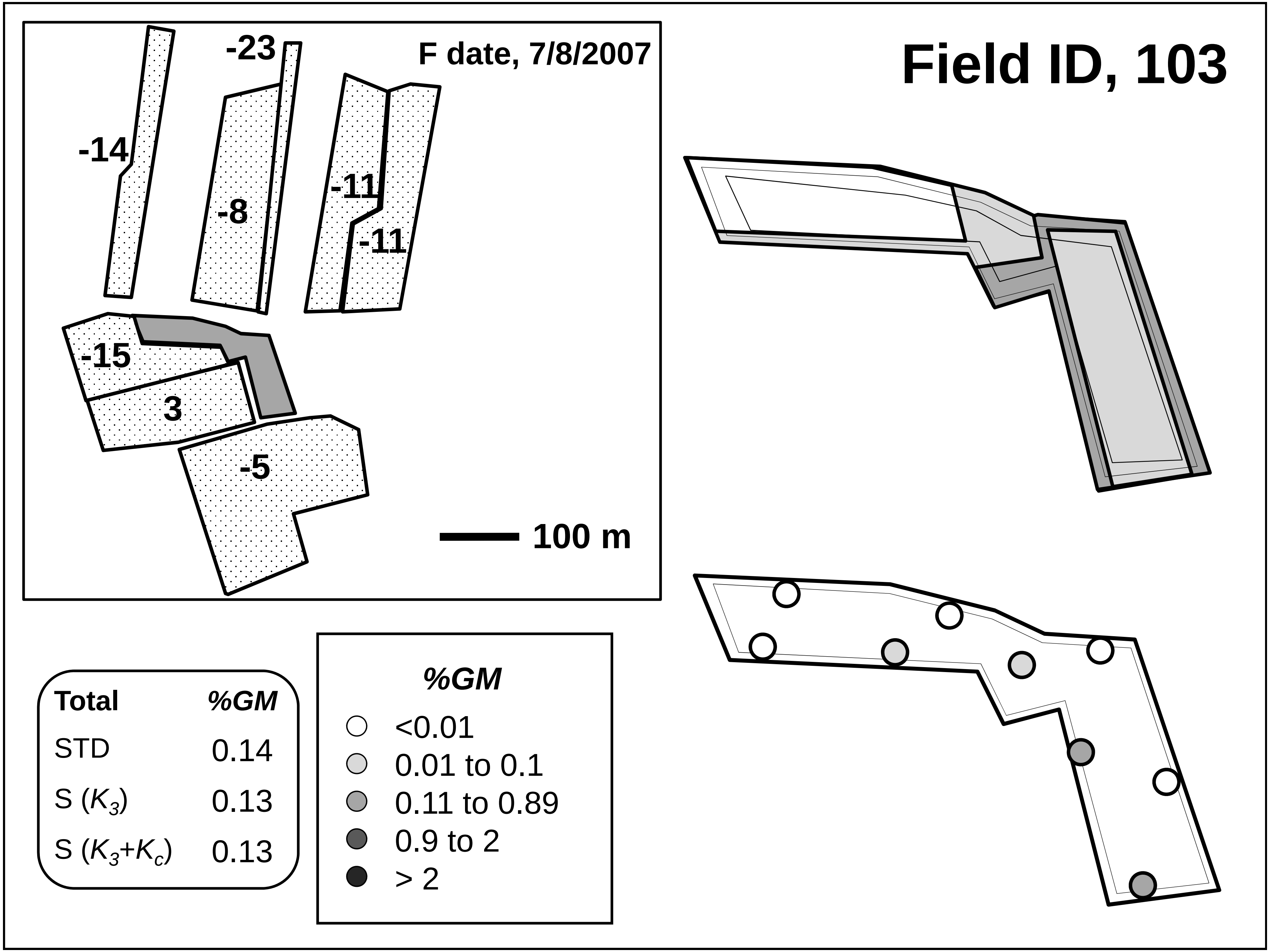
Field ID, 103
F date, 7/8/2007
-23
-14
-8
-11
-11
-15
3
-5
100 m
Total
%GM
STD
0.14
S (K_3)
0.13
S (K_3+K_c)
0.13
%GM
<0.01
0.01 to 0.1
0.11 to 0.89
0.9 to 2
> 2

Field ID, 1

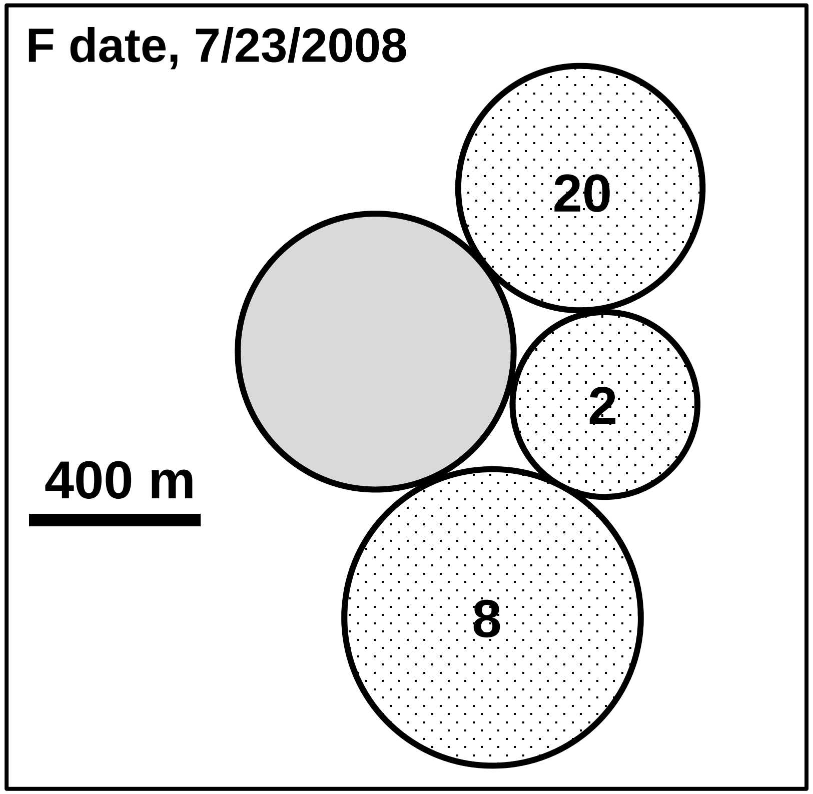

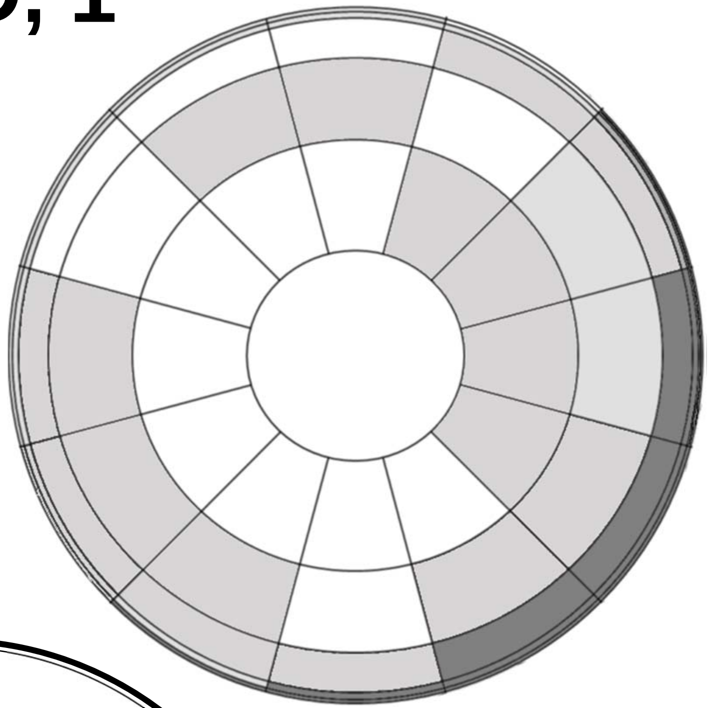

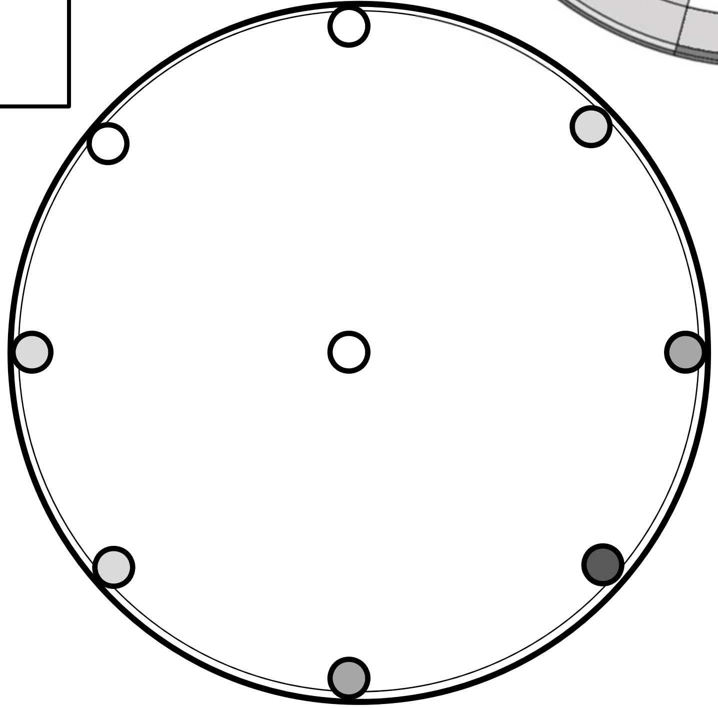

%GM

- <0.01
- 0.01 to 0.1
- 0.11 to 0.89
- 0.9 to 2
- > 2

**Total**	***%GM***
STD	0.04
S (K_3)	0.05
S (K_3+K_c)	0.05

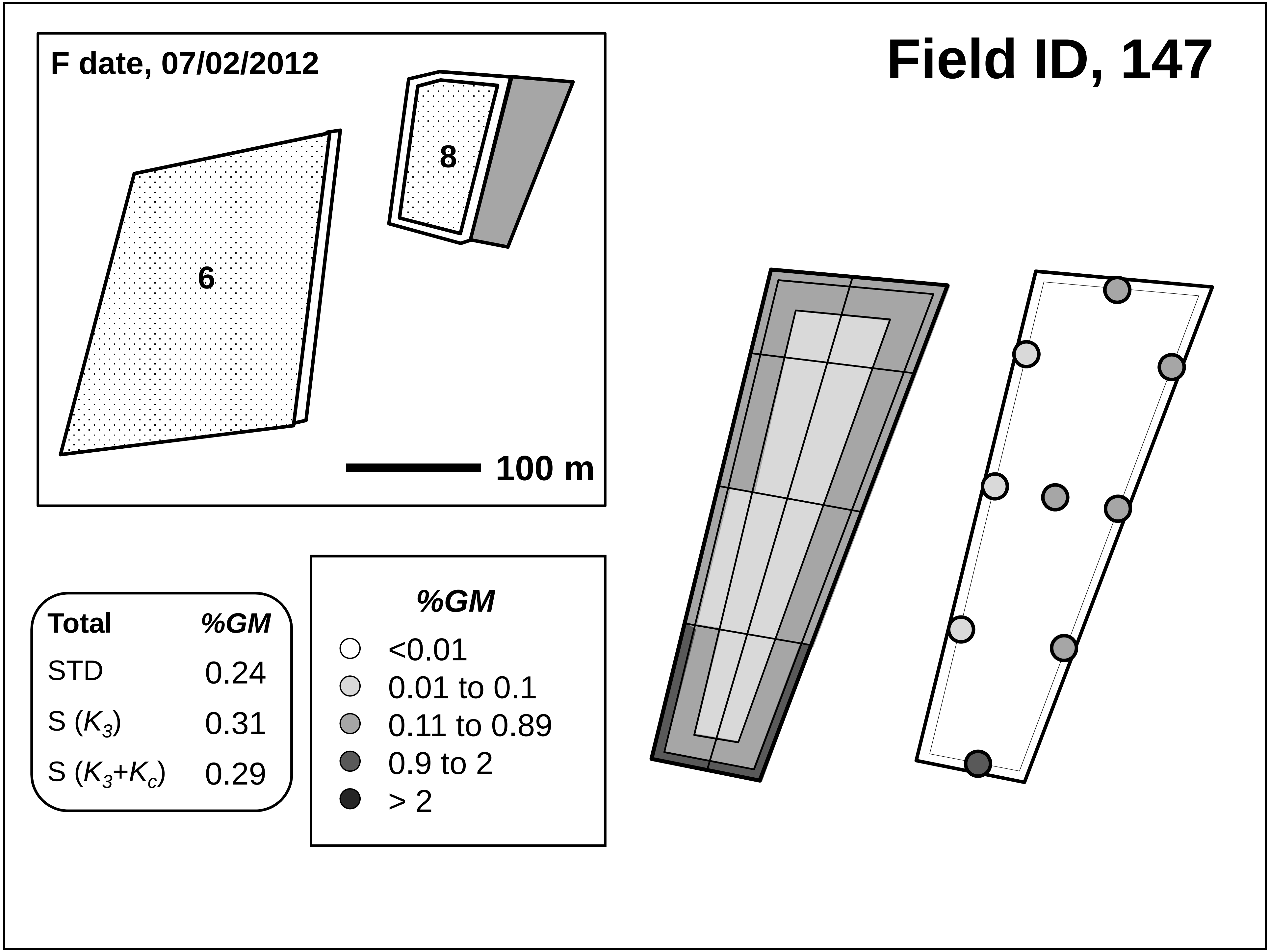
Field ID, 147
F date, 07/02/2012
8
6
100 m
Total %GM
STD 0.24
S (K_3) 0.31
S (K_3+K_c) 0.29
%GM
<0.01
0.01 to 0.1
0.11 to 0.89
0.9 to 2
> 2

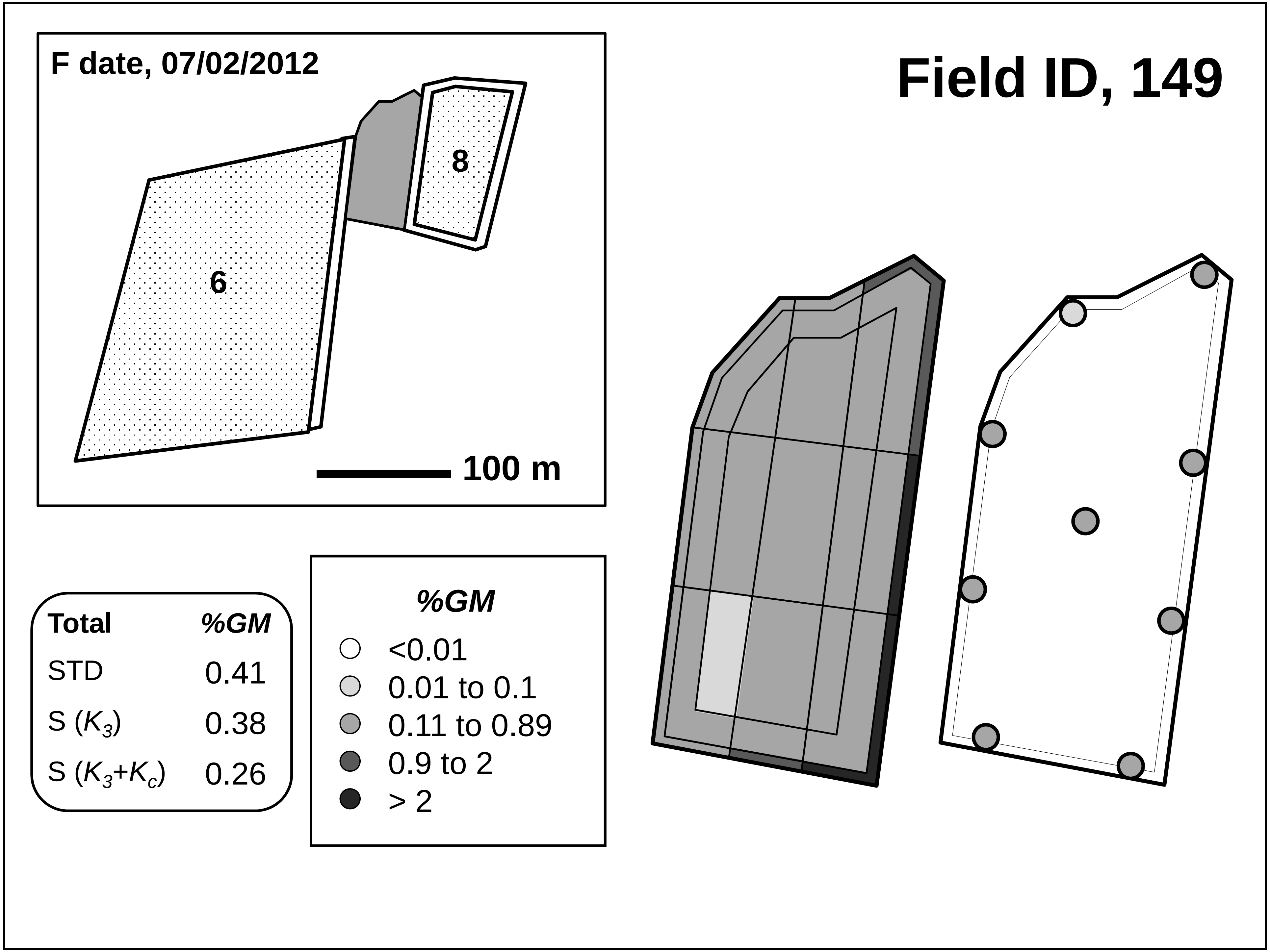

Field ID, 149
F date, 07/02/2012
8
6
100 m
Total
%GM
STD
0.41
S (K_3)
0.38
S (K_3+K_c)
0.26
%GM
<0.01
0.01 to 0.1
0.11 to 0.89
0.9 to 2
> 2

Field ID, 2A

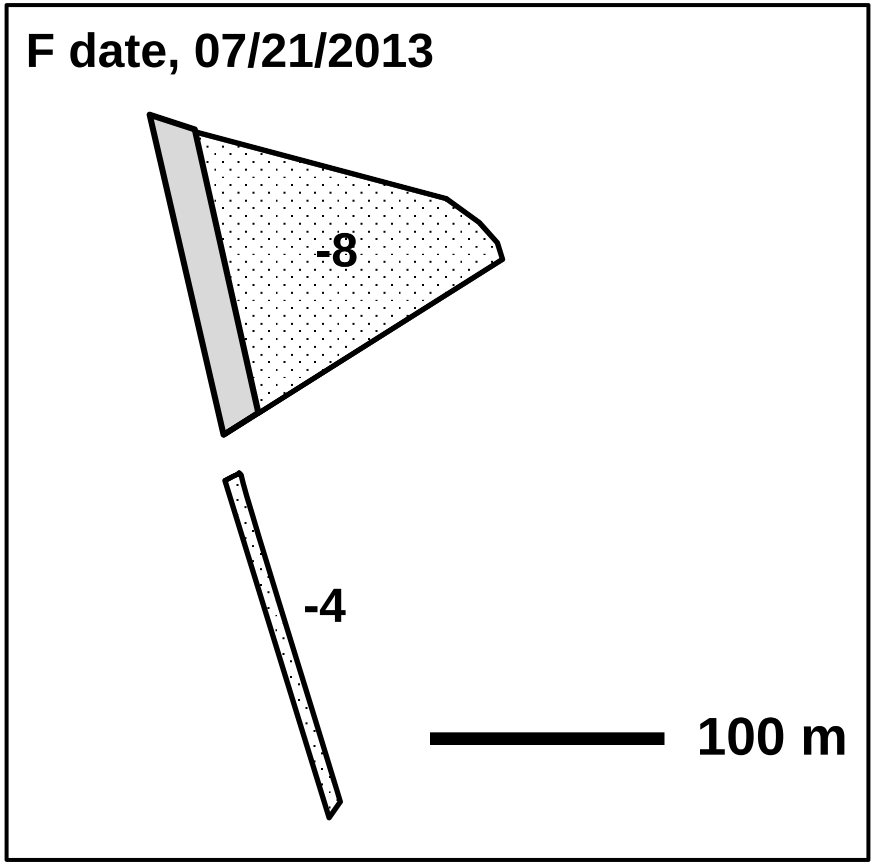

Total	*%GM*
STD	0.07
S (K_3)	0.01
S (K_3+K_c)	0.01

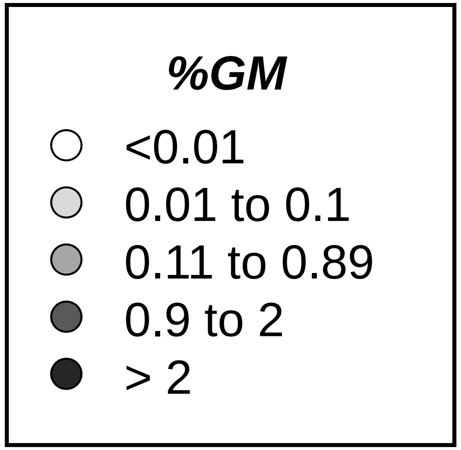

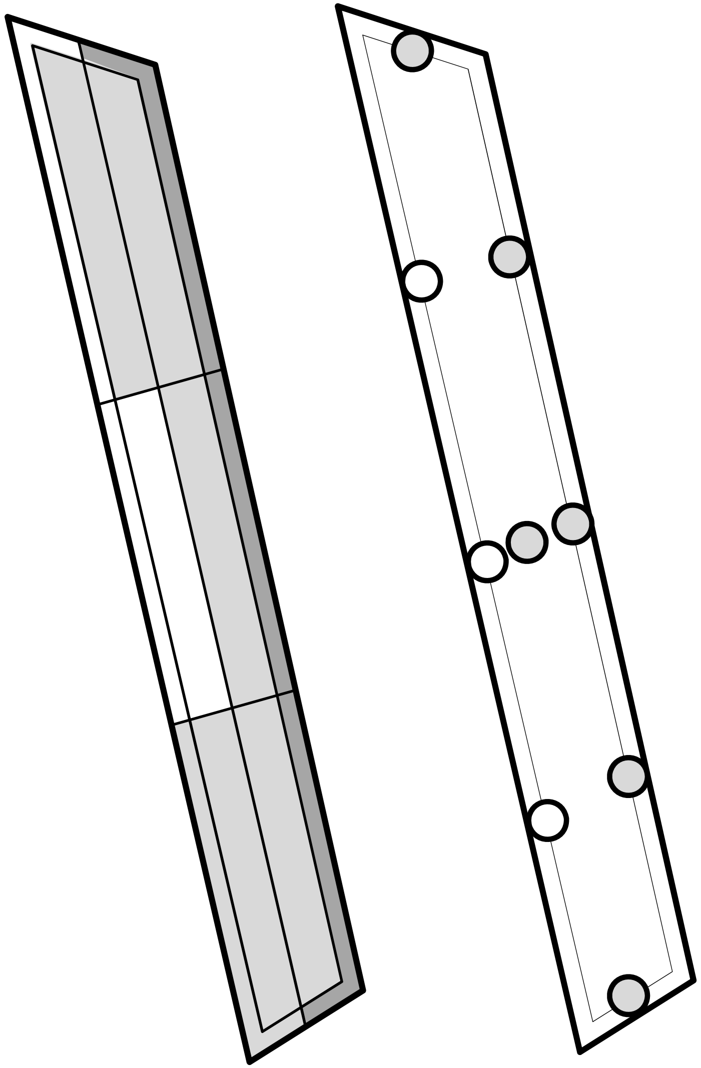

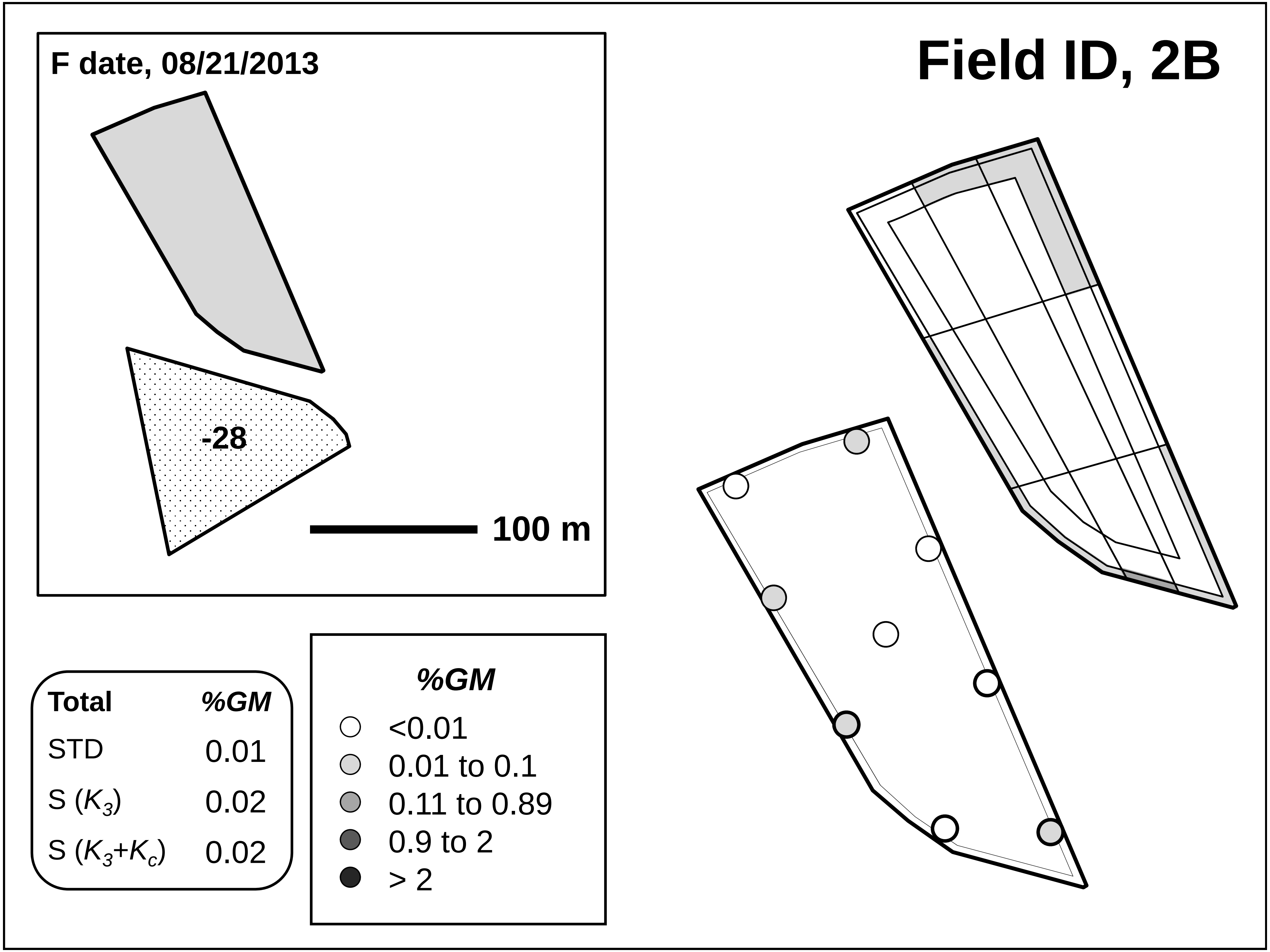
Field ID, 2B
F date, 08/21/2013
-28
100 m
Total %GM
STD 0.01
S (K_3) 0.02
S (K_3+K_c) 0.02
%GM
<0.01
0.01 to 0.1
0.11 to 0.89
0.9 to 2
> 2

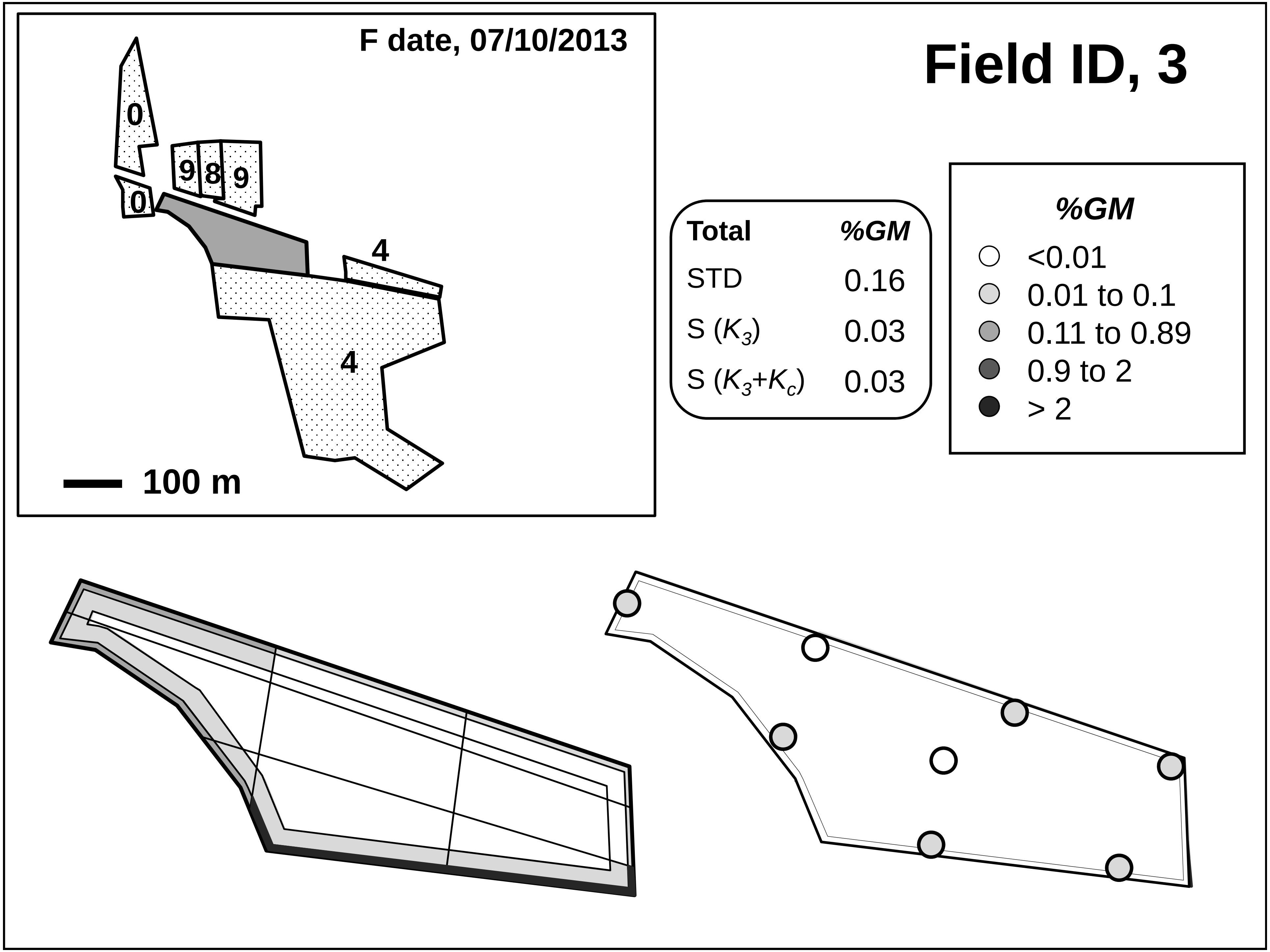
F date, 07/10/2013
0
0
9
8
9
4
4
100 m
Field ID, 3
Total %GM
STD 0.16
S (K_3) 0.03
S (K_3+K_c) 0.03
%GM
<0.01
0.01 to 0.1
0.11 to 0.89
0.9 to 2
> 2

Field ID, 4

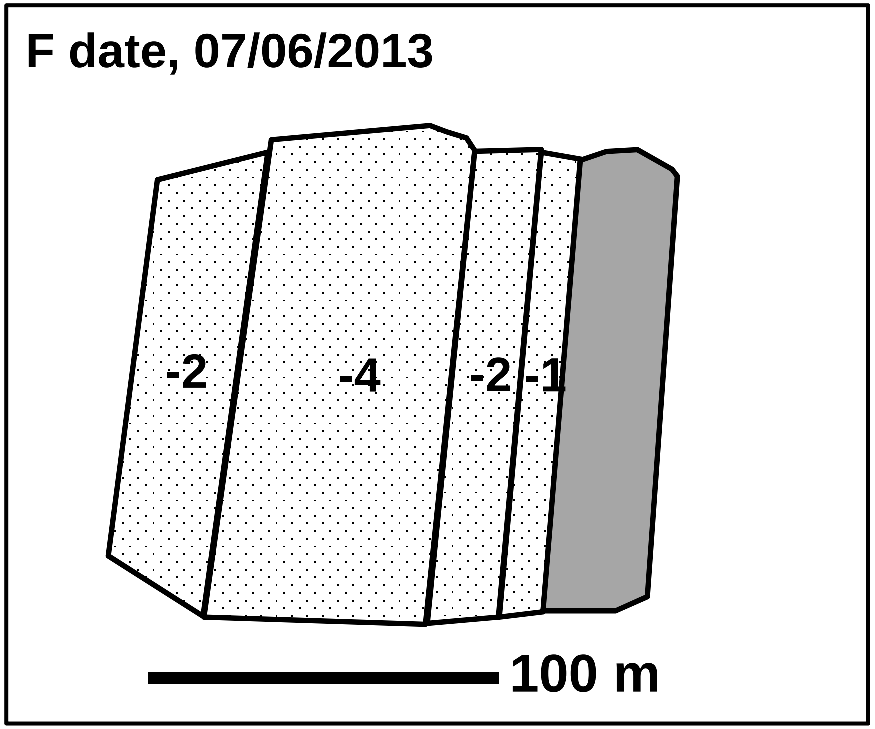

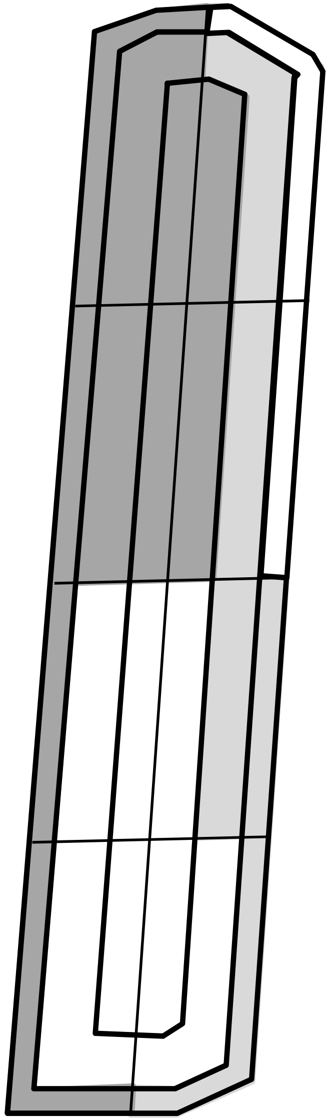

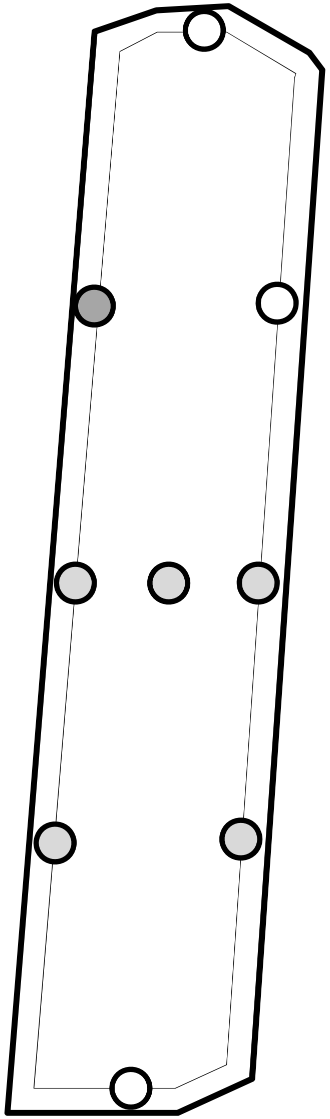

Total	*%GM*
STD	0.12
S (K_3)	0.05
S (K_3+K_c)	0.05

%GM

- <0.01
- 0.01 to 0.1
- 0.11 to 0.89
- 0.9 to 2
- > 2

Supplementary Figure 4. Adventitious GM contents in conventional maize agricultural fields monitored in the 2005 to 2013 seasons. For every receptor field, a map is displayed (upper-left side) showing the conventional field under study and the surrounding GM donor fields (up to a 120 m distance from the conventional field, represented in a dotted pattern). Central numbers correspond to the interval (in days) between male flowering of every donor field and female flowering of the receptor field (F date). Additional schemes of the receptor field represent: (i) Left or up, adventitious GM distribution in the conventional field measured using the standard approach. Samples were taken at the intersection of the lines drawn within the field under study (i.e. at d = 0, 3 and 10 m around the field, and the field center) and qPCR analyzed. Colors in the schemes represent *%GM* in the different subareas (i.e. means of qPCR values at the 4 vertices of each subarea). Overall *%GM* (STD) corresponds to the weighted mean of all subareas. (ii) Right or down, adventitious GM distribution in this conventional field measured using the simplified approach. The same color code is used to represent *%GM* values of K_C and the individual samples around the field. Overall *%GM* was calculated with K_3 and equation (2) [S(K_3)], and K_3, K_C and equation (4) [S(K_3+K_C)]. Individual analysis of the 8 periphery samples (represented in the scheme right or up) allows identifying the neighbor field causing adventitious GM occurrence.

Supplementary Figure 5

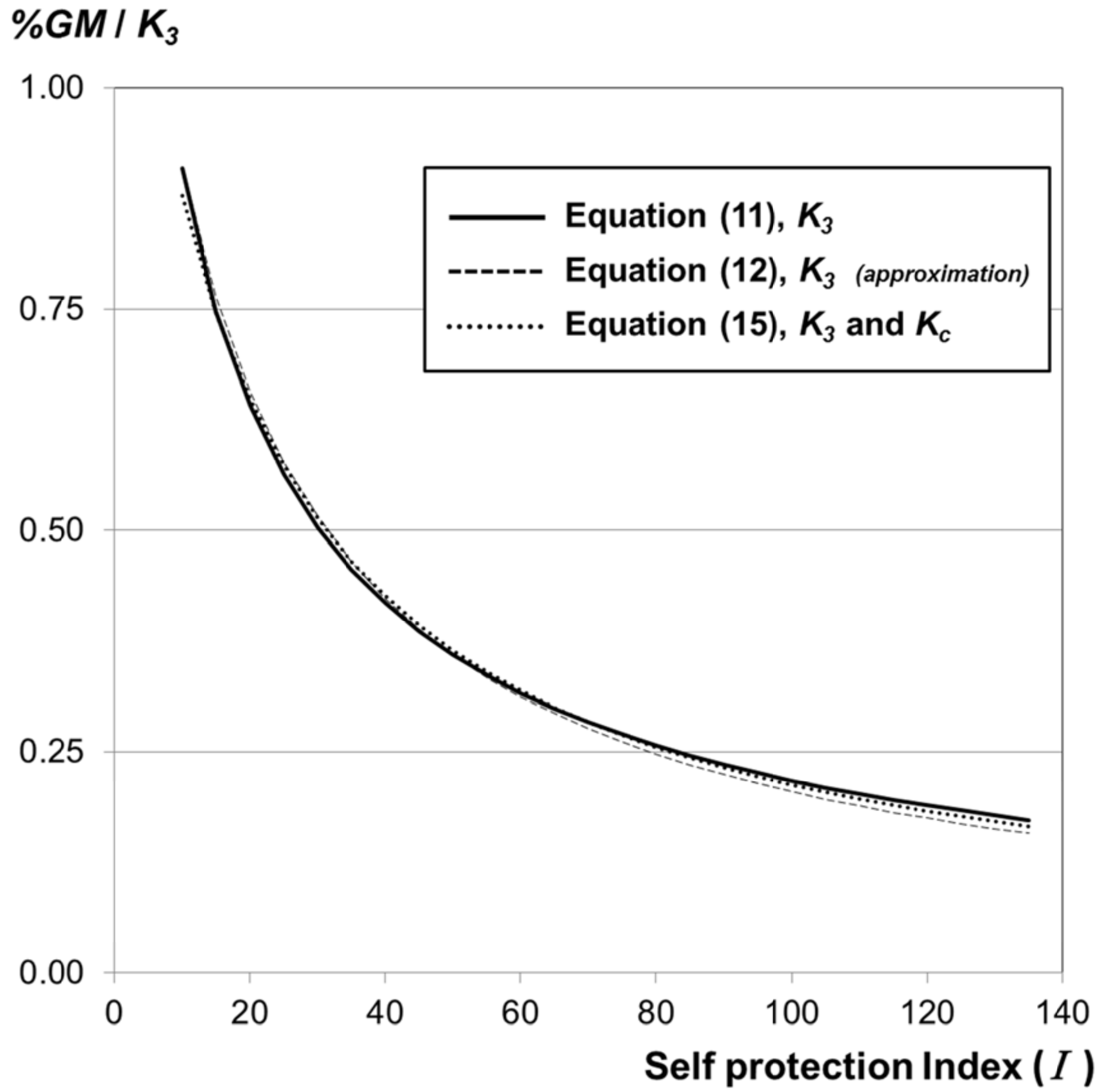

Supplementary Figure 5. Variation of *%GM* contents in conventional fields as a function of the I index. Represented are the values obtained by application of the model [equation (3) and the approximate equation (4), both based on K_3] and the estimation obtained by dividing the field into two concentric portions [equation (5), based on K_3 and K_c]. Note that in a perfectly square field, values obtained with equation (6) (not represented) exactly match those obtained with equation (5). All curves were normalized to achieve average K_3 = 1. For a field with e.g. I = 80, K_3 = 1 indicates 0.25% GM in the yield. Note that the aim of this comparison was not to show the agreement of the different equations to the reality but to prove coherence between the three formulae.

Supplementary Figure 6

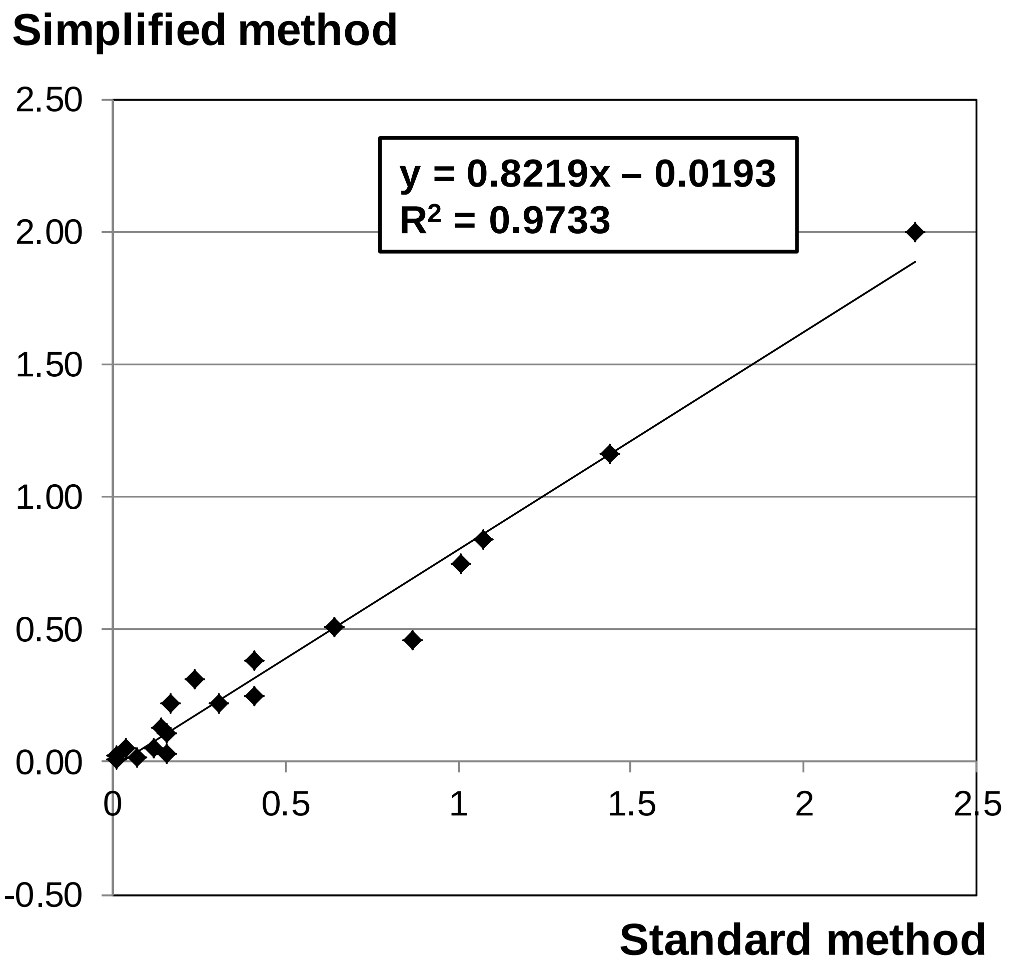

Supplementary Figure 6a. Comparison between the *%GM* in conventional maize fields as calculated on the basis of samples taken following the standard and the simplified sampling approaches. Every data-point corresponds to a field; and the (x,y) coordinates correspond to *%GM* percentages obtained using the standard and the simplified sampling methods. Inbox, regression curve and R^2 value.

Supplementary Figure 6

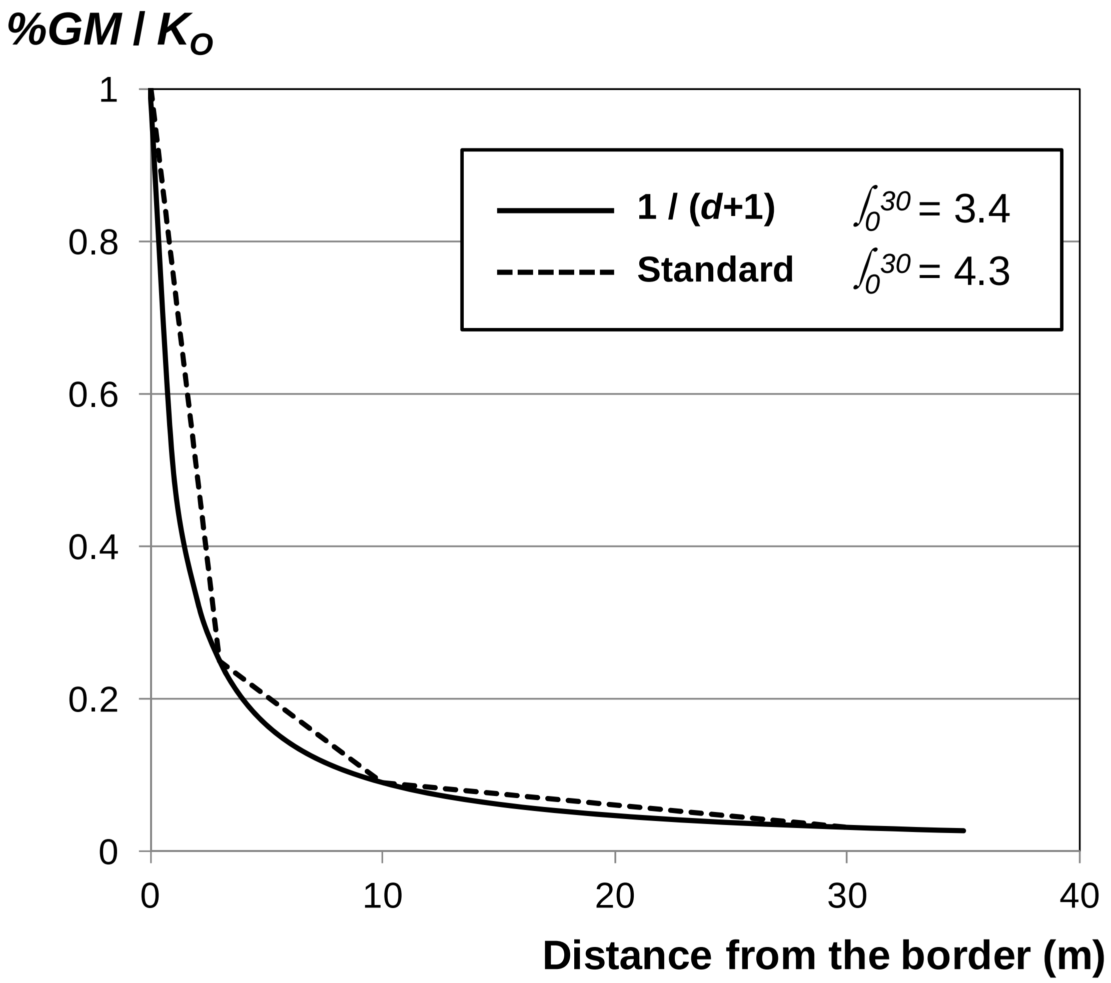

Supplementary Figure 6b. Distribution pattern of adventitious GM contents in conventional maize fields, either considering a hypothetical continuous curve (continuous line) and a discontinuous pattern reflecting the experimental calculation in the standard sampling method with samples at *d* = 0, 3, 10 and 30 m from the field borders (discontinuous line). The cross-fertilization diminution curve 1/*d*+1 is the mathematical fundament for the simplified approach. In contrast, the standard calculation gives every sub-area a GM value corresponding to the arithmetic mean of the values at the four vertices. In this example the area under the curve 1/*d*+1 in the *d* = 0 to 30 m range ($\int_0^{30}$) is 3.4; whereas that below the dotted line representing the standard sampling method is $\int_0^{30}$ = 4.3 (i.e. about 20% above the former value). The real distribution in a field is not continuous since maize rows are typically 0.75 m apart. Taking the maize row distance into account $\int_0^{30}$= 3.5, thus virtually the same as the 1/*d*+1 theoretical one.

Supplementary Figure 7

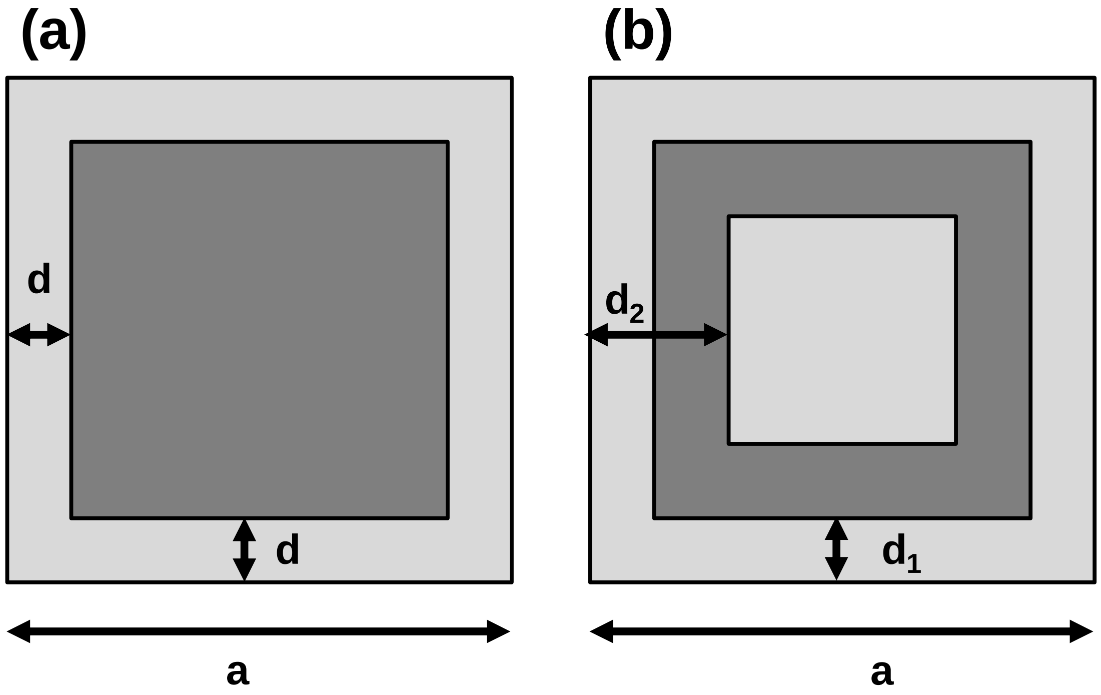

Supplementary Figure 7. Square fields with side *a*. **(a)** All points in the perimeter of the inner square are at a distance *d* from the field border. Therefore, the mean density at a distance *d* is the average density through this inner perimeter. **(b)** Square field with a concentric region (dark grey). Points in the most internal perimeter are at a distance d_2 from the field border, and points in the middle perimeter are at a distance d_1 from the field border.
